# Genomic phylogeny, taxonomic classification, genetic landscape, and antibiotic resistance of clinical *Stenotrophomonas* isolates

**DOI:** 10.3389/fmicb.2026.1854839

**Published:** 2026-07-13

**Authors:** Valery V. Shapovalova, Elvira R. Shaidullina, Natali V. Ivanchik, Igor V. Chebotar, Anna V. Mikotina, Aida N. Chagaryan, Elena Y. Skleenova, Ilya S. Azizov, Andrey V. Romanov, Andrey V. Dekhnich, Roman S. Kozlov, Mikhail V. Edelstein

**Affiliations:** Institute of Antimicrobial Chemotherapy, Smolensk State Medical University, Smolensk, Russia

**Keywords:** antibiotic resistance, beta-lactamases, genomic phylogeny, mobile genetic elements (MGEs), phosphoethanolamine transferases (PETs), plasmids, Stenotrophomonas, taxonomic classification

## Abstract

**Background:**

*Stenotrophomonas* spp. are increasingly recognized opportunistic pathogens characterized by intrinsic multidrug resistance. Despite recent taxonomic reclassification and the delineation of new species within the *S. maltophilia* complex (Smc), routine diagnostics often misidentify these species, and their distinctive antibiotic resistance traits and genetic makeup remain underexplored. We aimed to characterize the phylogenomic diversity, genotypic and phenotypic antibiotic resistance traits, virulence markers and horizontally transferrable elements in a large national cohort of clinical *Stenotrophomonas* isolates.

**Methods:**

We analyzed 323 clinical isolates from the Russian sentinel surveillance program (2002–2021). Analysis included MALDI-TOF MS identification, broth microdilution susceptibility testing to six agents [aztreonam-avibactam, colistin, levofloxacin, minocycline, tigecycline, and trimethoprim-sulfamethoxazole (TMP-SMX)], and long-read Nanopore WGS with hybrid polishing. We performed average nucleotide identity (ANI)-based classification, core- and accessory-genome comparisons, and comprehensive profiling of antibiotic resistance genes (ARGs), virulence factors (VFs), plasmids, and prophages.

**Results:**

While MALDI-TOF MS identified all isolates as *S. maltophilia*, ANI (≥95% threshold) and core-genome phylogeny revealed 17 distinct species: 52% *S. maltophilia* sensu stricto, 36.8% across eight other ICNP-named Smc species, and 11.2% within eight “novel” genomic lineages identified elsewhere. Pan-genome comprised 24,815 genes (9.6% core genes), with accessory genes showing robust species-level clustering in UMAP. TMP-SMX resistance was prevalent (40.2%) among all species, though only 13.1% of resistant isolates harbored *sul* genes. Intrinsic β-lactamase genes (*bla*_L1_- and *bla*_L2_-like) were ubiquitous but exhibited high sequence variability (up to 20.6% and 25.1%) and evidence of inter-species exchange. Phosphoethanolamine transferase genes (*mcr*-5- and *mcr*-8-like) were identified as intrinsic chromosomal components. Acquired ARGs were primarily associated with chromosomal integrative conjugative and mobilizable elements (ICEs/IMEs). Plasmids (2.5 to >334 kb) were identified in 8% of isolates across 10 species, including a cluster of eight nearly identical replicons distributed among five species despite lacking canonical mobilization genes. Prophages were ubiquitous (median: 4 per genome), dominated by *Caudoviricetes* and *Inoviridae*. Several VFs linked to motility, capsule formation, and invasion/toxicity were universally present across the genus.

**Conclusion:**

Our findings demonstrate that current routine diagnostics fail to resolve the significant taxonomic diversity within clinical *Stenotrophomonas* isolates, leading to widespread species misidentification. Integrating the genomic and phenotypic insights from this study into routine surveillance is essential to overcome these diagnostic limitations and to modernize the clinical management of infections caused by this formidable genus.

## 1 Introduction

*Stenotrophomonas* is a diverse and environmentally widespread bacterial genus increasingly recognized for its clinical significance, particularly in compromised individuals and patients with underlying conditions such as cystic fibrosis (Izydorczyk et al., 2023; Guo et al., 2025). Originally classified under *Pseudomonas*/*Xanthomonas, Stenotrophomonas* was recently redefined into the order *Lysobacterales* and family *Lysobacteraceae* based on phylogenomic evidence, with modern genomic studies revealing widespread species misidentification and driving proposals for new species and genera (Ma et al., 2025; Kumar et al., 2019; Bansal et al., 2023). Among the genus *Stenotrophomonas, Stenotrophomonas maltophilia* has been the most commonly isolated species from human infections and has emerged as a formidable opportunistic and nosocomial pathogen due to its intrinsic resistance to multiple classes of antibiotics, limited treatment options, and ability to persist in hospital environments (Li et al., 2024; Tamma et al., 2024; Gawande and Sande, 2024; Niemiec et al., 2024; Mojica et al., 2022). Recent studies have revealed that *S. maltophilia* represents a complex of distinct phylogenetic lineages, which under contemporary genosystematic criteria have been delineated and proposed as separate novel species (Gröschel et al., 2020; Mustapha et al., 2022; Wang et al., 2024; Ortiz Álvarez et al., 2025).

Despite its clinical relevance, the taxonomy of *Stenotrophomonas* remains underexplored in routine diagnostics (Yu and Wang, 2024). Accurate identification and differentiation of *Stenotrophomonas* species pose significant challenges, primarily due to the insufficient discriminatory power of conventional biochemical tests, which often yield overlapping metabolic profiles among related and even distant species of this genus (Fluit et al., 2022). Efforts to improve resolution with MALDI-TOF MS, a widely adopted rapid identification tool in clinical microbiology, have also encountered notable difficulties with *Stenotrophomonas*. Although MALDI-TOF MS consistently outperforms biochemical systems at genus-level identification, its performance in resolving *Stenotrophomonas* at the species level remains suboptimal (Gautam et al., 2017; de Miranda et al., 2023; Vasileuskaya-Schulz et al., 2011).

Historically, *S. maltophilia* has been characterized by its formidable intrinsic resistance to a broad spectrum of antimicrobial agents, a trait largely dictated by its complex natural resistome. This inherent resistance is primarily driven by the co-expression of the chromosomal β-lactamases L1 and L2, alongside a diverse array of multidrug efflux pumps, which collectively render most conventional β-lactams, aminoglycosides, and quinolones ineffective. However, the clinical challenge is increasingly compounded by reports of acquired resistance to the old and newly developed antimicrobial agents (Wang et al., 2018). Resistance to trimethoprim-sulfamethoxazole (TMP-SMX), traditionally considered a first-line therapy for *S. maltophilia* infections, is of particular concern, with reports of regional variability in resistance rates and increasing frequencies in some parts of the world (Gales et al., 2019; Sader et al., 2025; Wang et al., 2025). The mechanisms underlying this resistance are multifactorial, often involving mutations in the target genes, increased antibiotic efflux, or acquisition of mobile genes (Sánchez and Martínez, 2018; Ochoa-Sánchez et al., 2024). Despite this escalating threat, the distribution and clinical impact of these resistance traits remain largely unexplored within the context of the recently updated taxonomy of *Stenotrophomonas*. As many isolates previously identified as *S. maltophilia* are now recognized as distinct species within the *S. maltophilia* complex (Smc), there is a critical need to re-evaluate how the intrinsic and acquired resistance determinants are partitioned across this newfound taxonomic diversity to inform more precise diagnostic and therapeutic strategies.

Beyond chromosomal determinants, the genomic plasticity of *Stenotrophomonas* is driven by a diverse mobilome, although the relative contribution of specific mobile genetic elements (MGEs) to horizontal gene transfer (HGT) remains unevenly characterized. Current evidence indicates that while *Stenotrophomonas* lacks the high plasmid burden seen in other gram-negative bacteria, it frequently harbors T4SS-type integrative and conjugative elements (ICEs) and integrative mobilizable elements (IMEs), particularly those of the *clc*-type and *Tn4371*-like families, which mediate the acquisition of *sul1* and other resistance markers (Sum et al., 2025; Ryan et al., 2009; Boncompagni et al., 2025). However, significant knowledge gaps persist regarding the “cryptic” plasmidome of *Stenotrophomonas*; while some long-read sequencing studies detected no plasmids in completed genomes of environmental and human isolates representing major Smc phylogenetic lineages (Thant et al., 2026a), others identified replicons typically lacking homology to known incompatibility groups, with nearly identical plasmids occurring across divergent lineages despite absent or incomplete canonical mobilization machinery (Thant et al., 2026a). Furthermore, while prophages are ubiquitous and contribute to intra-species diversification, their specific role in disseminating functional resistance or virulence cargo across the recently reclassified species boundaries of the Smc has not been systematically addressed (Fang et al., 2023; Lee et al., 2014). These open questions regarding non-canonical transfer mechanisms highlight the need for high-resolution genomic studies to resolve the pathways of gene flux in this genus.

In this study, we present a comprehensive phenotypic and genomic analysis of 323 clinical *Stenotrophomonas* spp. isolates collected from hospitalized patients across Russia over a 19-year period, combining antimicrobial susceptibility testing with whole-genome sequencing to characterize the genomic diversity, taxonomic distribution, and patterns of resistance and virulence within this national cohort. Our findings reveal substantial genomic heterogeneity and unmask a high frequency of species misidentification in clinical diagnostics, while elucidating the interplay between phenotypic resistance and the functional genetic landscape of the genus; collectively, these results underscore the necessity of integrating genomic insights into routine surveillance to refine the clinical management of *Stenotrophomonas* infections.

## 2 Methods

### 2.1 Bacterial strains

Clinical isolates of *Stenotrophomonas* spp. (*n* = 323) and accompanying metadata were collected at 76 hospitals in 42 cities across Russia in 2002–2021 as part of the national sentinel surveillance program of antimicrobial resistance in bacterial pathogens isolated from hospitalized patients (Kuzmenkov et al., 2021). The isolates were recovered from clinical specimens (sputum, bronchoalveolar lavage, endotracheal aspirate, blood, wound discharge, urine, etc.) of 301 patients with various acute infections, and from respiratory specimens of 22 cystic fibrosis (CF) patients with chronic infections. Only one isolate per patient/case of infection was included, screening and environmental samples were spared. The isolates were referred to the central surveillance laboratory and were maintained in 30% glycerol supplemented broth in deep freezing at −80 °C until analysis. All the isolates included in this study were preliminary identified at the central laboratory using two MALDI-TOF MS platforms in parallel, Microflex LT-MALDI Biotyper System (Bruker Daltonics, Bremen, Germany) and Autof MS2000 (Autobio Diagnostics, Zhengzhou, China).

### 2.2 Antibiotic susceptibility testing

Minimum inhibitory concentrations (MICs) of six antibiotics and combinations: aztreonam-avibactam (4 mg/L fixed avibactam concentration), colistin, levofloxacin, minocycline, tigecycline, and trimethoprim-sulfamethoxazole (TMP-SMX; 1:19 fixed ratio), were determined using the reference broth microdilution method in Mueller-Hinton II Broth (Liofilchem, Roseto degli Abruzzi, Italy) according to ISO 20776-1:2019 (International Organization for Standardization, 2019). *Escherichia coli* ATCC 25922, *E. coli* NCTC 13846, *Klebsiella quasipneumoniae* ATCC 700603, and *Pseudomonas aeruginosa* ATCC 27853 were used as quality control strains for antibiotic susceptibility testing. Isolates were categorized as wild-type (WT, antibiotic susceptible) or non-wild-type (non-WT, antibiotic resistant) based on the European Committee on Antimicrobial Susceptibility Testing (EUCAST) MIC epidemiological cut-off values (ECOFFs; European Committee on Antimicrobial Susceptibility Testing, 2026a).

### 2.3 Whole-genome sequencing

Genomic DNA (gDNA) was extracted from homogeneous bacterial colonies of pure overnight cultures using a DNeasy Blood & Tissue Kit (Qiagen, Hilden, Germany) according to the manufacturer's protocol for extraction of gDNA from gram-negative bacteria. DNA purity and concentration were determined using a Nanophotometer/Fluorometer DS-11FX+ and a dsDNA High Sensitivity Kit (DeNovix, Wilmington, DE, USA), and DNA integrity was assessed using a TapeStation 4150 Agilent, Santa Clara, CA, USA) according to the manufacturer's instructions. All gDNA samples were sequenced using long-read nanopore sequencing on a GridION (Oxford Nanopore Technologies, Oxford, UK). The long-read libraries were prepared using either a Ligation Sequencing Kit (SQK-LSK109) with Native Barcoding Expansion (EXP-NBD104) or Ligation sequencing—Native Barcoding Kits (SQK-NBD112.24 or SQK-NBD114.24) and were then run on FLO-MIN109 (R9.4.1) or FLO-MIN114 (R10.4.1) flow cells according to the manufacturer's protocols. Base calling of raw reads was performed using Dorado v.0.9.0 and v.0.9.1 basecaller (Oxford Nanopore Technologies, 2026a) with the super-accurate model (SUP). In addition, 92 isolates selected to represent different phylogenomic clades and subclades identified by interim clustering analysis of long-read genomic sequences were sequenced using short-read sequencing on a DNBSEQ-G99 (MGI Tech, ShenZhen, China) to obtain high-quality hybrid genome assemblies. The short-read libraries were prepared using a MGIEasy Fast PCR-FREE FS Library Prep Set and were run on a PE300 flow cell (DNBSEQ-G99RS High-throughput Sequencing Set App-D FCL PE300).

### 2.4 Genome assembly and quality control

FastQC v0.12.0 (Andrews, 2010) was used to analyze the quality of short sequencing reads of the studied isolates, as well as reads from public genomes, and pycoQC (Leger and Leonardi, 2019) was used to analyze the nanopore reads. The genomes were assembled using the Dragonflye v.1.2.1 pipeline (Petit, 2024). Briefly, long reads were filtered using Nanoq v.0.10.0 (Steinig and Coin, 2022), retaining only the reads with a minimum length of 500 bp and a minimum q-score of 10. Short reads, if available, were trimmed using fastp v.0.23.4 (Chen et al., 2018). Flye v2.9.3-b1797 (Kolmogorov et al., 2019) with option “–nanohq” was used to produce assemblies. Each assembly was then polished with nanopore reads using Medaka v1.11.3 (Oxford Nanopore Technologies, 2026b) with models r941_min_sup_g507 and r941_e81_sup_g514 for the genomes sequenced on R9.4.1 flow cells and using Medaka v2.0.1 with option “–bacteria” for the genomes sequenced on R10.4.1 flow cells. Circular contigs were reoriented to begin with the replication initiator gene using Dnaapler v.0.7.0 (Bouras et al., 2024a). Finally, 92 hybrid assemblies were polished using trimmed short reads and Polypolsh v0.6.0 (Bouras et al., 2024b) with “–careful” option. Quast v5.3.0 (Gurevich et al., 2013) and CheckM v.1.2.3 (Parks et al., 2015) were used to estimate genome quality and to determine assembly statistics. The final data set contained 323 newly sequenced genomes with a median coverage depth of 229 × (range 58–398x) for long reads and 149x (range 76–297x) for short reads (Supplementary Table S1). Median *Q*-scores of long reads obtained from R9.4.1 and R10.4.1 flow cells were, respectively, 13.47 (range 12.5–17.12) and 21.19 (range 20.57–22.3). Median lengths of long reads obtained from R9.4.1 and R10.4.1 flow cells were, respectively, 5,461 bp (range 425 bp−10,803 bp) and 3,754 bp (range 303 bp−5048 bp). All assemblies were assessed for completeness (range 91.02–100, median 100) and contamination (range 0–4.14, median 0.29) using CheckM (Parks et al., 2015). The majority of assemblies (*n* = 287, 88.3%) consisted of one contig (range 1–6) and median N50 was 4,745,889 bp (range: 4,227,729–5,094,380 bp).

### 2.5 Taxonomic assignment

Reference genomes of type strains of *Stenotrophomonas* species validly published under the International Code of Nomenclature of Prokaryotes (ICNP) were downloaded from NCBI databases (February 2025) using NCBI Datasets v16.27.1 (O'Leary et al., 2024) run with flag “–from-type,” and at least one reference genome of each species was used for species identification of clinical isolates. Additionally, at least one genome from each of the 23 *Stenotrophomonas* genomic lineages as previously defined by Gröschel et al. (2020) was included. FastANI v.1.34 (Jain et al., 2018) was employed to calculate the pairwise average nucleotide identity (ANI). A ≥95% ANI was used as the cut-off to define a bacterial species. Firstly, the genomes of the isolates from this study were compared to those of the type strains from the NCBI data set and were assigned to species validly published under the ICNP. The genomes that remained unassigned to species were further analyzed using the Type Strain Genome Server (TYGS) (Meier-Kolthoff et al., 2022) and, finally, if the ANI values against type strains of all species published under ICNP were below the 95% cut-off, were compared to reference genomes from the international data set (Gröschel et al., 2020) and assigned to genomic lineages named according to the original nomenclature used by Gröschel et al. (2020). The accession numbers of selected reference genomes (*n* = 25) used in this study are found in Supplementary Table S2.

Pairwise distances between the genomes were calculated by subtracting the percentage ANI values from 100 and dividing the results by 100, and were then used to perform a hierarchical clustering using hclust with the average linkage method (method = “average”) implemented in R v.4.1.2. The analysis workflow was implemented as a reproducible pipeline using Nextflow (Di Tommaso et al., 2017), with the code available at https://github.com/valery-shap/tax-pipeline. A dendrogram and a heatmap were plotted using heatmap.2 function of the gplot package.

### 2.6 Core and pan-genome analysis

Genomes were annotated using Prokka v. 1.14.6 (Seemann, 2014) with default options. Panaroo v1.5.0 (Tonkin-Hill et al., 2020) was used to generate a gene presence-absence matrix with the default settings and the -a core flag to generate a core gene alignment. A core gene phylogeny was constructed from the filtered core gene alignment file using the IQ-Tree software v2.0.4 (Minh et al., 2020; Kalyaanamoorthy et al., 2017) with the GTR substitution model and ultrafast bootstrapping (1,000 bootstraps). The phylogenetic tree was visualized using Microreact (Argimón et al., 2016). A gene presence-absence binary matrix was used to calculate the Russell-Rao distance between the pairs of genomes as implemented in the Scikit-learn v1.6.1. Genes that were present in only one genome were removed from analysis. Then, the UMAP v0.5.7 (Ehiro, 2023) algorithm was used to project the full multidimensional binary matrix into two-dimensional space.

### 2.7 Genome annotation

#### 2.7.1 Antimicrobial resistance genes (ARGs)

Antimicrobial resistance genes were identified using AMRFinderPlus v.4.0.19 (Feldgarden et al., 2021) with “-p,” “-n,” “-gff,” “-a prokka,” and “-plus,” flags. This tool combines BLASTP, BLASTX and HMMER to identify the resistance determinants. Genes encoding Sme efflux transporters were identified with Abricate v.1.0.0 (Seemann, 2024) using a custom database compiled from the CARD database (Alcock et al., 2023) and the K279a reference genome annotation (Crossman et al., 2008). Additionally, the assemblies were screened for the presence of intrinsic L1- and L2-like beta-lactamase genes using BLASTN (Camacho et al., 2009) and a custom database of *bla*_L1_ and *bla*_L2_ gene sequences downloaded from BLDB (Beta-lactamase database) (Naas et al., 2017). The identified *bla*_L1_- and *bla*_L2_-like gene sequences were first compared using BLAST to assess similarity and were then aligned with MAFFT v7.525 (Katoh and Standley, 2013). The resulting alignments were used to infer phylogenetic trees with IQ-TREE v2.4.0 (Minh et al., 2020; Kalyaanamoorthy et al., 2017) using the TIM3+F+I+G4 model for *bla*_L1_ and the TN+F+G4 model for *bla*_L2_. Branch support was assessed using ultrafast bootstrap analysis with 1,000 replicates. To assess species-specific clustering, monophyly of lineages was evaluated using the check_monophyly() function from the ETE3 Python package (v3), based on tip labels corresponding to species assignments. Aligned *bla*_L1_- and *bla*_L2_-like sequences were further analyzed for intragenic recombination using Recombination Detection Program (RDP) v5.84 (Martin et al., 2021). Genes encoding phosphoethanolamine transferases (PETs) were extracted from all genomes based on Prokka annotations. Annotated PETs shorter than 100 amino acids were excluded from further analysis. Reference MCR protein sequences (*n* = 118) were downloaded from the AMRFinderPlus database. All PET and reference MCR amino acid sequences were aligned using MAFFT v7525 (Katoh and Standley, 2013) with default parameters. A maximum-likelihood phylogeny was inferred in IQ-TREE v2.4.0 (Minh et al., 2020) using ModelFinder (Kalyaanamoorthy et al., 2017), which selected the JTT+F+R6 substitution model. Branch support was assessed with 1,000 ultrafast bootstrap replicates. Additionally, the genomic context of *eptA* genes was analyzed in all hybrid assemblies, which revealed the presence of a single copy of each *eptA* gene type. Genes located within a ±4-gene window flanking each *eptA* locus were extracted and clustered using the DIAMOND v2.1.16 clustering module (Buchfink et al., 2021; Saad et al., 2025), and unique neighborhood variants were identified. Genes encoding the trimethoprim and sulfamethoxazole target proteins (FolA and FolP) and the regulators of the SmeDEF and SmeVWX multidrug efflux systems (SmeT and SmeRv) were also screened for structural mutations potentially associated with trimethoprim–sulfamethoxazole resistance. The *folA* (SMLT_RS03880), *folP* (SMLT_RS08345), *smeT* (SMLT_RS19375), and *smeRv* (SMLT_RS08825) gene sequences were extracted from all genomes using BLASTN (Camacho et al., 2009) searches with homologous genes from the *S. maltophilia* K279a reference genome as queries. The extracted nucleotide sequences were translated and aligned using MAFFT v7.525 (Katoh and Standley, 2013).

#### 2.7.2 Virulence factors

Virulence factors were identified using the Abricate tool (Seemann, 2024) with the Virulence Factor Database (VFDB) database (Liu et al., 2022) applying a minimum identity and coverage threshold of 60%. Additionally, a curated database of experimentally characterized *Stenotrophomonas maltophilia* virulence-associated genes was screened using Abricate (70% identity). The database included genes encoding extracellular proteases (*stmPr1* [SMLT_RS03270], *stmPr2* [SMLT_RS04110], and *stmPr3* [SMLT_RS20900]), determinants previously implicated in biofilm formation and surface colonization (*smf-1* [SMLT_RS03355], *rmlA* [SMLT_RS03090], *rpfF* [SMLT_RS10745], and *spgM* [SMLT_RS23485]), and the flagellin gene *fliC* (SMLT_RS11075), which encodes the major structural component of the flagellar filament. Sequences possessing the Functions of Sequences of Concern (FunSoCs) and putatively associated with pathogenicity, were identified using the SeqScreen tool (Balaji et al., 2022).

#### 2.7.3 Phages, plasmids, and integrative conjugative elements (ICEs)

Phage and plasmid contigs were identified using the geNomad tool (Camargo et al., 2024). Plasmid replicons were further detected using Abricate (Seemann, 2024) with the PlasmidFinder database (Carattoli and Hasman, 2020). MOB-suite software tools were used to classify plasmids and predict their transferability (Robertson and Nash, 2018). Clustering of plasmid contigs was performed using the Pling tool (Frolova et al., 2024). Analysis of integrative and conjugative/mobilizable elements (ICEs/IMEs) was conducted using ICEberg 3.0 (Wang et al., 2024), IntegronFinder 2.0 (Néron et al., 2022), and ISFinder (Siguier, 2006).

## 3 Results

### 3.1 Sources of bacterial isolates

A total of 323 clinical isolates of the genus *Stenotrophomonas*, collected between 2002 and 2021 as part of the antimicrobial resistance (AMR) sentinel surveillance program (Kuzmenkov et al., 2021), from 76 hospitals across 41 cities in Russia and one city in Kazakhstan, were included in this study (Supplementary Table S3). Of these, 301 (93.19%) were obtained from patients with various acute infections and 22 (6.81%) from cystic fibrosis (CF) patients with chronic respiratory infections. The majority of isolates originated from infections of respiratory tract (201/323, 62.23%), followed by the abdominal cavity (36/323, 11.15%), blood stream (33/323, 10.22%), skin and soft tissues (24/323, 7.43%), urinary tract (22/323, 6.81%), bones and joints (5/323, 1.55%), and the central nervous system (2/323, 0.62%; Supplementary Table S4). All isolates were considered clinically significant according to established criteria (Miller et al., 2018; Hauser et al., 2011). Despite subsequent genomic reclassification discussed later in the manuscript, all isolates were initially identified as *Stenotrophomonas maltophilia* with reliable species-level scores on both Autobio Autof and Bruker Biotyper MALDI-TOF MS platforms.

### 3.2 Phylogenomics and taxonomic identification of Stenotrophomonas spp.

**Figure 1:**
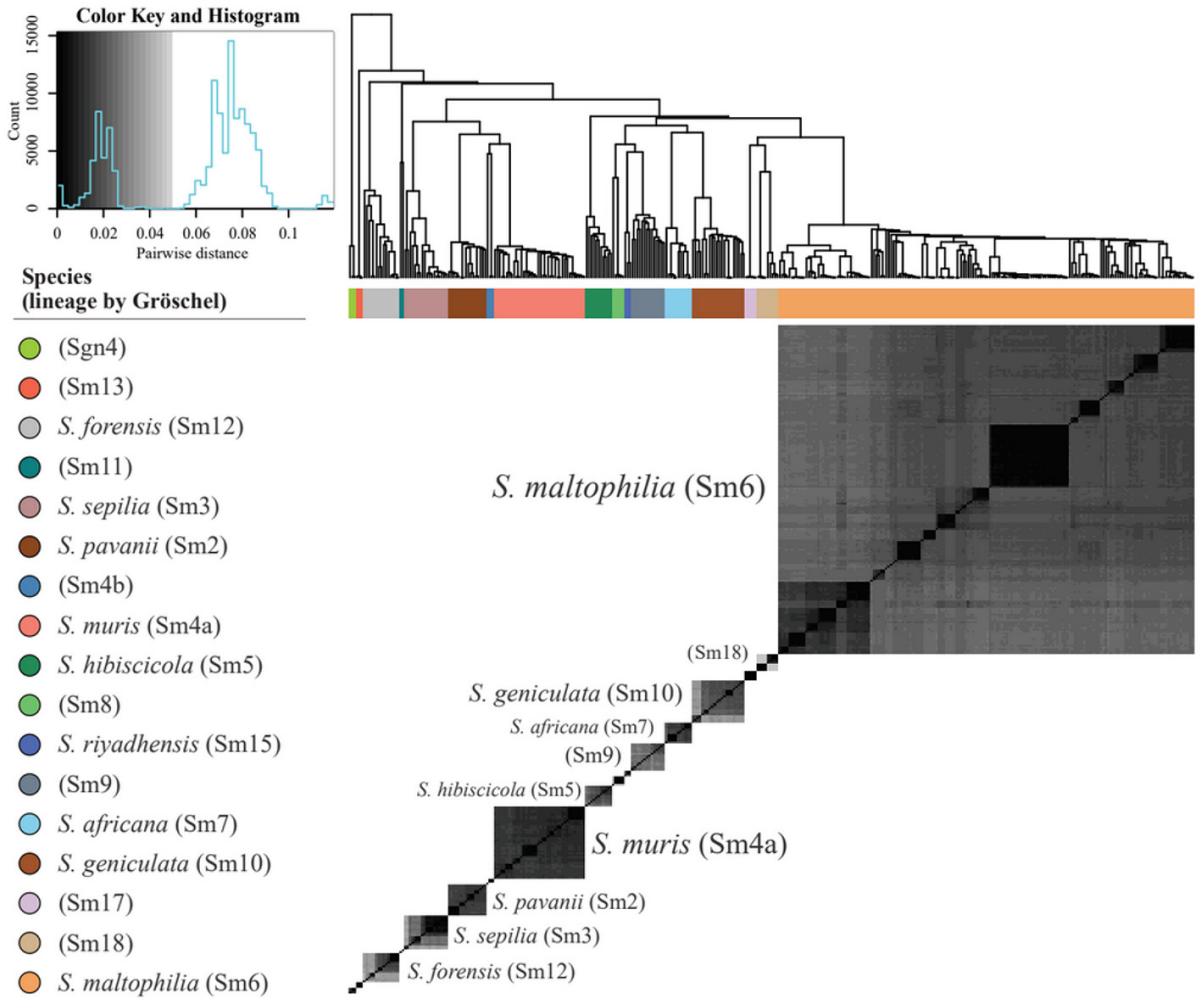
Hierarchical clustering dendrogram and heatmap based on FastANI values for isolates from this study and reference strains. Gray shading indicates similarity values ≥0.95, with darker shades indicating higher similarity. Major species and lineages are indicated.

Pairwise comparisons of average nucleotide identity (ANI) values (range: 88%−100%) were performed for 323 newly sequenced clinical isolate genomes, together with 25 reference genomes, encompassing type strains of validly published named species and representatives of major *Stenotrophomonas* species lineages previously described (Gröschel et al., 2020), hereinafter jointly referred to as genomic species (Supplementary Figure S1). A 95% ANI threshold was applied to delineate species (Figure 1).

This analysis identified 17 distinct genomic species among the isolates. The majority (287/323, 88.85%) were assigned to nine validly published named species, with just over half (168/323, 52%) classified as *S. maltophilia*. The remaining 36 (11,15%) isolates were delineated into eight genomic lineages, as described by Gröschel et al. (2020), based on closest ANI matches (≥95%) to their respective representative genomes. Of the 17 species identified, 16 were putatively classified as members of the *S. maltophilia* complex based on an ANI threshold of ≥90% among isolates (*n* = 321), whereas one species, Sgn4 (*n* = 2), was more distantly related to the others, showing ANI values of 88.09%−89.08%. All genomes fulfilled the recently established criterion of average amino acid identity (AAI) ≥88% for inclusion within the genus *Stenotrophomonas* (Chauviat et al., 2025), with a minimum AAI value of 90.44% observed between the two genomes of *S. muris* (Sm4a, isolate 77931) and Sgn4 (isolate 2201). Besides *S. maltophilia*, six most abundant species: *S. muris* (Sm4a), *S. geniculata* (Sm10), *S. sepilia* (Sm3), *S. forensis* (Sm12), *S. pavanii* (Sm2), and Sm9, included more than 10 isolates each. Although isolates of these species were recovered from diverse infection sites, the respiratory tract constituted the principal source for each species, and no significant differences in site distribution were observed between species (Supplementary Table S4). All but two isolates recovered from the respiratory tract of CF patients belonged to *S. maltophilia*. The latter two CF isolates were identified as *S. muris* and Sm17.

### 3.3 General genome characteristics, core-genome, and pan-genome

The genome sizes of the analyzed *Stenotrophomonas* isolates ranged from 4.3 Mb (Sgn4 isolate 112478) to 5.12 Mb (*S. maltophilia* isolate 32257; Supplementary Figure S2A). The GC content showed relatively low variation across genomes, with values ranging from 65.86% (Sm9, isolate 93052) to 67.16% (*S. pavanii* isolate 68302; Supplementary Figure S2B). Pan-genome analysis identified a total of 24,815 genes, including 2,391 core genes present in more than 99% of genomes, and 20,574 cloud genes found in fewer than 15% of genomes. The large number of cloud genes highlights the extensive genomic plasticity of *Stenotrophomonas* species implicated in human infections.

**Figure 2:**
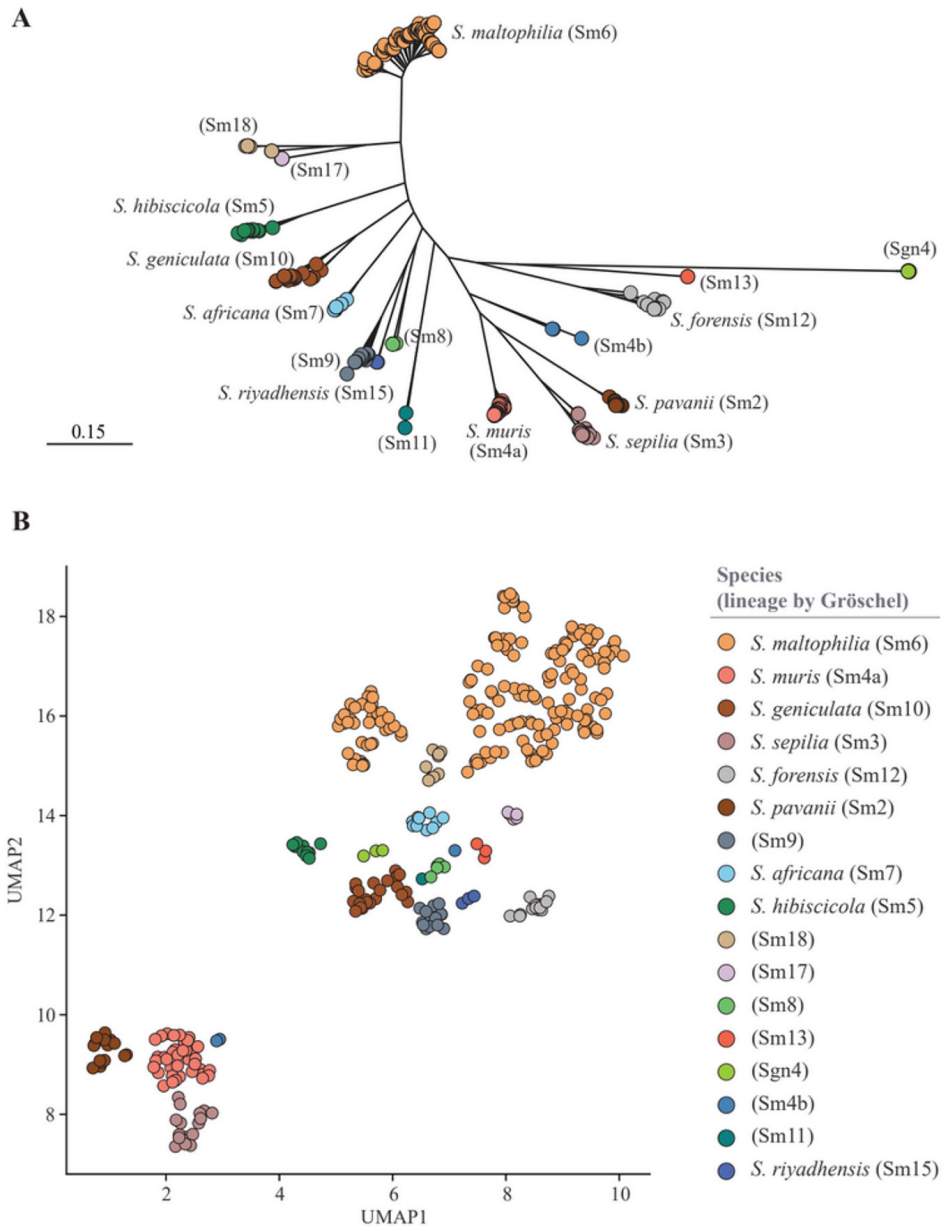
Core-genome and pan-genome analysis. **(A)** Maximum-likelihood phylogeny inferred from polymorphic sites in the core-gene alignment. The tree was built using 1,000 ultrafast bootstrap replicates. Nodes are colored according to species as determined by the ANI analysis described above. **(B)** UMAP visualization of accessory gene content across genomes. Each point represents a genome colored according to species/lineage.

A phylogenetic tree was constructed based on a concatenated core-gene alignment of 2,391 genes from 348 genomes (323 sequenced in this study and 25 reference genomes). The resulting maximum-likelihood tree (Figure 2A) revealed deep phylogenetic divisions that closely corresponded to the species-level groupings defined by ANI.

Consistent with the core-genome phylogeny, accessory gene content also showed clear structure in UMAP space (Figure 2B). Genomes belonging to the same species lineage generally formed coherent groups, indicating species-specific accessory genome composition.

### 3.4 Antibiotic resistance phenotypes

**Figure 3:**
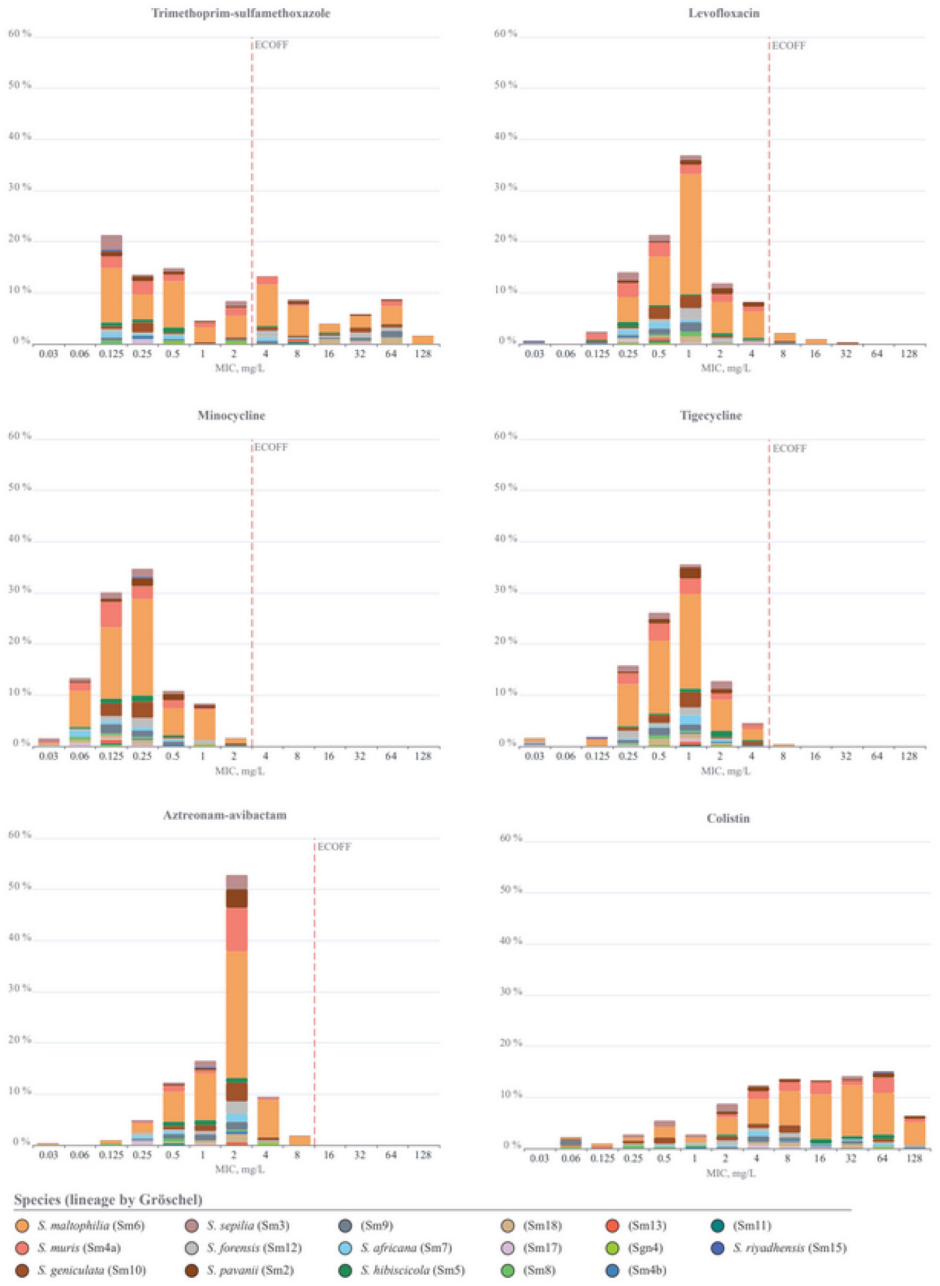
Minimum inhibitory concentration (MIC) distributions of antibiotics across *Stenotrophomonas* species. Vertical dashed lines denote epidemiological cut-off values (ECOFFs).

The antibiotic susceptibility testing of studied isolates revealed unimodal MIC distributions for all antibiotics except trimethoprim-sulfamethoxazole (TMP-SMX; Figure 3).

The mode MIC, MIC_50_, and MIC_90_ values of levofloxacin (1, 1, and 4 mg/L), minocycline (0.25, 0.25, and 0.5 mg/L), tigecycline (1, 1, and 2 mg/L), and aztreonam-avibactam (2/4, 2/4, and 4/4 mg/L) were all below the ECOFF breakpoints. In contrast, TMP-SMX exhibited a non-unimodal MIC distribution, with MIC values broadly dispersed across isolates from all abundant species (MIC_50_: 1/19 mg/L; MIC_90_: 32/608 mg/L), and did not display a species-specific resistance pattern (Supplementary Figure S3). All isolates were categorized as wild-type susceptible to aztreonam-avibactam and minocycline. Tigecycline resistance was detected in a single isolate of *S. maltophilia* (0.31%), levofloxacin resistance in 11 isolates from four species (3.41%), and TMP-SMX resistance in 130 isolates from 13 species (40.24%). Thus, resistance to levofloxacin and TMP-SMX was not restricted to *S. maltophilia* but observed across multiple species without a statistically significant species association. Notably, isolates from CF patients exhibited significantly higher levofloxacin resistance than those from non-CF patients (4/22, 18.18% vs. 7/301, 2.33%, odds ratio: 9.33, *P* = 0.0039, Fisher's exact test). In contrast, resistance to TMP-SMX was less frequent among CF isolates (7/22, 31.82% vs. 123/301, 40.86%, odds ratio: 0.68), although this difference was not statistically significant (*P* = 0.8595). Colistin exhibited a broad MIC distribution across all *Stenotrophomonas* species, with MIC_50_ and MIC_90_ values reaching 16 mg/L and 64 mg/L, respectively. Overall, 88.54%, 85.75%, 76.77% and 64.08% of isolates demonstrated resistance at colistin concentrations of 0.5, 1, 2, and 4 mg/L, respectively. Therefore, although clinical MIC breakpoints and epidemiological cut-off values (ECOFFs) for colistin against *Stenotrophomonas* have not been defined by EUCAST or CLSI, most isolates would be classified as resistant according to pharmacokinetic/pharmacodynamic (PK/PD)-based thresholds established for other bacterial species (Tran et al., 2016).

### 3.5 Antibiotic resistance genes (ARGs)

#### 3.5.1 β-Lactamase genes

Genes encoding L1- and L2-like β-lactamases (*bla*_L1_- and *bla*_L2_-like), which are considered intrinsic to *S. maltophilia*, were detected in almost all isolates and had conserved positions in the chromosomes of all species.

Specifically, *bla*_L1_-like genes were present in 322 of 323 genomes, although 3 isolates of *S. sepilia* had truncated sequences of *bla*_L1_. The only genome lacking *bla*_L1_ was a *S. forensis* isolate 97088. *bla*_L2_-like genes were found in all genomes.

Additionally, a BLAST search was performed to assess the inter- and intra-species similarity of *bla*_L1_- and *bla*_L2_-like gene sequences among 92 *Stenotrophomonas* isolates representing different species, for which high-quality hybrid genome assemblies were generated from combined long- and short-read sequencing data. Both *bla*_L1_-like and *bla*_L2_-like genes exhibited significant sequence variability among different species, with median nucleotide sequence identities of 88.81% and 87.9%, respectively. For *bla*_L1_-like genes, the lowest sequence identities were observed between isolate 2201 of Sgn4 and those of *S. forensis* (isolate 120459, 79.61%) and Sm4b (isolate 85239, 79.38%). For *bla*_L2_-like genes, the most divergent sequences (74.87% identity) were found between isolate 125580 of *S. forensis* and six isolates of *S. muris*. The *bla*_L1_-like gene sequences were highly conserved (>98% identity) within each species of *S. maltophilia, S. pavanii, S. africana*, and Sm8, but showed lower intraspecies conservation (< 90% identity) within *S. sepilia*, Sm4b, Sm9, and Sm18. Similarly, *bla*_L2_-like genes exhibited variable levels of nucleotide sequence conservation within species, with the highest identities (>98%) observed in *S. geniculata, S. pavanii*, Sm8, and Sm9, and the lowest (< 80%) in *S. forensis, S. muris*, and *S. sepilia* (Table 1).

**Table 1:** Intra-species sequence similarity of *bla*_L1_- and *bla*_L2_-like genes.

Species (Gröschel lineage)^*^	*N*	*bla*_L1_ sequence similarity, median (range), %	Monophyly	*bla*_L2_ sequence similarity, median (range), %	Monophyly
*S. maltophilia* (Sm6)	36	99.08 (98.4–100.0)	Yes	99.18 (92.33–100.0)	No
*S. muris* (Sm4a)	14	98.97 (97.47–100.0)	Yes	99.34 (75.7–100.0)	No
*S. sepilia* (Sm3)	6	86.83 (83.97–100.0)	No	76.63 (76.34–100.0)	No
*S. forensis* (Sm12)	5	95.78 (95.24–97.54)	Yes	97.04 (76.02–99.34)	No
*S. geniculata* (Sm10)	5	97.19 (94.62–98.97)	Yes	99.34 (98.25–99.89)	Yes
*S. pavanii* (Sm2)	5	98.39 (98.05–100.0)	Yes	98.47 (98.36–100.0)	Yes
*S. africana* (Sm7)	4	99.54 (99.2–99.89)	Yes	97.53 (95.25–100.0)	No
(Sm18)	3	88.8 (88.8–100.0)	Yes	96.27 (94.63–96.71)	Yes
(Sm9)	3	93.36 (89.7–94.17)	No	98.52 (98.14–98.58)	Yes
(Sm4b)	2	87.62 (87.62–87.62)	No	97.27 (97.27–97.27)	No
(Sm8)	2	100.0 (100.0–100.0)	Yes	100.0 (100.0–100.0)	No
*S. hibiscicola* (Sm5)	2	94.29 (94.29–94.29)	Yes	89.39 (89.39–89.39)	No

^*^The species represented by only one hybrid genome assembly are excluded.

Phylogenetic analysis of *bla*_L1_- and *bla*_L2_-like genes was generally consistent with the patterns observed in sequence similarity comparisons, supporting strong intra-species conservation for several taxa. Consistent with the high sequence identities observed within *S. maltophilia, S. muris, S. geniculata, S. pavanii*, and *S. africana*, *bla*_L1_-like genes formed well-supported monophyletic clades in these species (Supplementary Figure S4A). Monophyly was also retained for less-represented species, such as *S. hibiscicola, S. forensis*, Sm8, and Sm18. In contrast, species exhibiting high *bla*_L1_ sequence divergence—*S. sepilia*, Sm4b, and Sm9—displayed a polyphyletic origin of *bla*_L1_-like genes. Subsequent analysis with the Recombination Detection Program (RDP) identified likely interspecies recombination events that produced chimeric *bla*_L1_ genes in six *S. sepilia* isolates, two *S. hibiscicola* isolates, and one *S. riyadhensis* isolate (Supplementary Table S5).

The *bla*_L2_ phylogeny showed less congruence with genome-based phylogeny and taxonomic assignment (Supplementary Figure S4B). Monophiletic origin of *bla*_L2_-like genes was supported only for *S. pavanii, S. geniculata*, Sm8, Sm9, and Sm18. Other species, including *S. maltophilia*, failed to form monophyletic clades. Recombination analysis (RDP) produced strong signals consistent with interspecies transfer and intragenic recombination affecting *bla*_L2_ in at least 13 isolates from six species (*S. africana, S. geniculata, S. hibiscicola, S. sepilia*, Sm9, and Sm13), originating from both polyphyletic and monophyletic clades (Supplementary Table S6).

The genes of acquired molecular class D penicillinases, *bla*_OXA − 1_, *bla*_OXA − 2_, *bla*_OXA − 392_ (*bla*_OXA − 1_-like), and a new *bla*_OXA_ variant (83.01% identical to *bla*_OXA − 42_-family gene, GenBank AccAP035784:1942355–1943224) were identified in six genomes, including three *S. maltophilia*, two *S. pavanii*, and one *S. geniculata*. Additionally, one *S. maltophilia* (isolate 149490) carried extended-spectrum β-lactamase (ESBL) gene cassette *bla*_GES − 7_, along with the *aac*(*6*′*)-Ib* and *qacE*Δ*sul1* gene cassettes, within a chromosomally embedded class 1 integron associated with an 84,972-bp integrative and mobilizable element (IME).

#### 3.5.2 Phosphoethanolamine transferase (PET) genes

Prompted by recent reports identifying phosphoethanolamine transferase (PET) genes homologous to mobile colistin resistance (*mcr*) genes in sporadic *Stenotrophomonas* isolates (Xie et al., 2021; Li et al., 2019; Gaballa et al., 2023), all genomes within this study were systematically screened for PETs. All identified PETs were chromosomally encoded and clustered phylogenetically into two lineages: MCR-5-like and MCR-8-like. These exhibited low-to-moderate amino acid identity to reference MCR proteins (40.94%−56.80% to MCR-5.1–5.5 and 51.52%−63.68% to MCR-8.1–8.5; Supplementary Figure S5).

**Figure 4:**
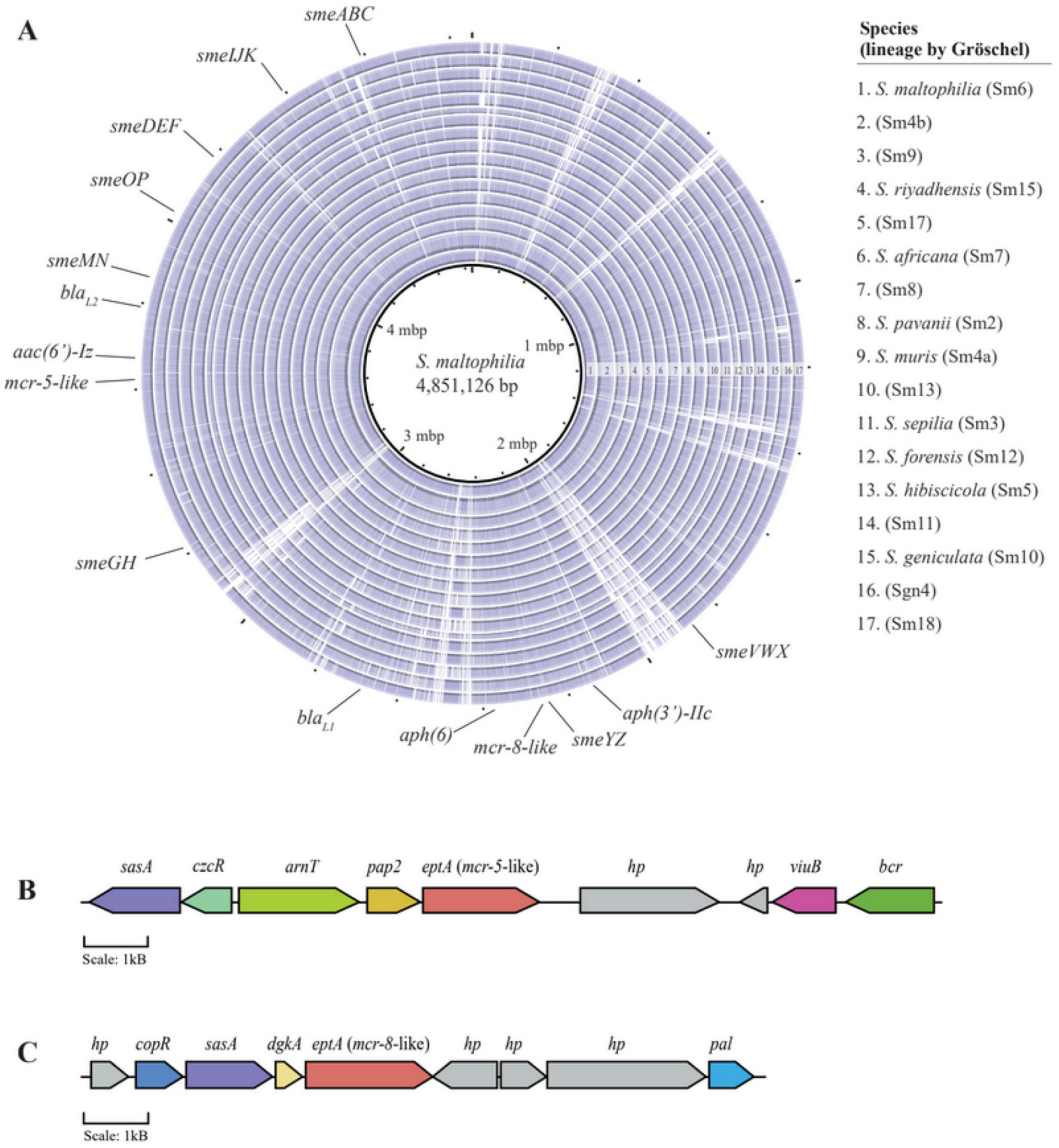
**(A)** BRIG comparison of chromosomes from representative isolates of 17 *Stenotrophomonas* species illustrating the genomic positions of major antibiotic resistance genes. **(B, C)** Typical genetic context of chromosomal *mcr*-5-like **(B)** and *mcr*-8-like **(C)** genes.

All genomes contained one or more PET genes of the *mcr*-5-like lineage; 17 genomes contained two, and one *S. maltophilia* isolate (55650) contained three. Genomic context analysis revealed a conserved structure associated with members of the *mcr*-5-like lineage across most *Stenotrophomonas* species (Figures 4A, B; Supplementary Figure S6A).

The upstream region (positions −4 to −1) was characterized by the sequential arrangement of *sasA* (adaptive-response sensory kinase), *czcR* (transcriptional regulator), *arnT* (undecaprenyl-phosphate arabinosyl transferase), and conserved *pap2* gene (PAP2 family phosphatase) immediately preceding the *mcr*-5-like gene. Downstream (positions +1 to +4), the typical organization comprised two conserved hypothetical protein genes, followed by *viuB* (vibriobactin utilization protein) and *bcr* (bicyclomycin resistance protein) genes. A subset of genomes deviated from this conserved structure due to insertions of mobile genetic elements. Insertion sequences from the IS3 and IS110 families were detected at various downstream positions (Supplementary Figure S6A).

PET genes from the *mcr*-8-like lineage were present in 85.45% of genomes (276/323), being nearly ubiquitous in *S. maltophilia, S. muris, S. geniculata, S. pavanii, S. africana, S. hibiscicola*, Sm18, Sm17, Sm13, and Sm11, but less common in *S. sepilia* and Sm9 and not detected in *S. forensis*, Sm8, Sm4b, or *S. riyadhensis*. The genomic context of *mcr*-8-like genes exhibited high conservation, typically comprising the arrangement: *hp* (conserved hypothetical protein 3)—*copR* (copper-responsive transcriptional activator)—*sasA* (adaptive-response sensory kinase)—*dgkA* (diacylglycerol kinase)—*mcr*-8-like—*hp* (M14 family metallocarboxypeptidase)—*hp* (conserved hypothetical protein)—*hp* (conserved hypothetical protein)—*pal* (peptidoglycan-associated lipoprotein) (Figures 4A, C; Supplementary Figure S6B).

#### 3.5.3 Other antibiotic resistance genes

The efflux operons associated with multidrug resistance—*smeDEF, smeYZ, smeIJK, smeOP*-*tolCsm, emrABC, smeU1VWU2X, smeGH*, and *smeMN*—were consistently present across genomes of all species, whereas *smeABC* was detected in most isolates but absent from all isolates of Sm8, Sm9, and Sgn4, as well as from a few isolates of *S. geniculata* and Sm4b (Supplementary Table S3).

Genes encoding aminoglycoside-modifying enzymes (AGMEs), including the intrinsic *S. maltophilia* aminoglycoside phosphotransferase gene *aph(6)*, were detected in all isolates across all *Stenotrophomonas* species. Another phosphotransferase gene, *aph(3*′*)-IIc*, was likewise present in all genomes except one *S. muris* isolate, four Sm18 isolates, and two Sgn4 isolates. In addition, 15 distinct acquired AGME genes were identified in 134 isolates representing 10 species.

The sulfonamide resistance genes *sul1* (*n* = 14) and *sul2* (*n* = 3) were found in 17 of 130 (13.1%) co-trimoxazole-resistant isolates belonging to *S. maltophilia* (*n* = 10), Sm9 (*n* = 4), *S. pavanii* (*n* = 2), and *S. geniculata* (*n* = 1).

Additionally, all genomes were screened for structural variations in the trimethoprim and sulfamethoxazole target proteins (FolA and FolP) and in the regulators of the SmeDEF and SmeVWX multidrug efflux systems (SmeT and SmeRv), which have been implicated in TMP-SMX resistance in the literature. The highest structural diversity was detected in FolP, which comprised 107 distinct allelic variants with variation at 192 amino-acid positions. FolA, SmeT, and SmeRv displayed 86, 71, and 29 protein variants, respectively, with variation observed at 81, 95, and 157 amino-acid positions (Supplementary Table S3). Whereas, diversity in FolA, FolP, and SmeT was mainly attributable to numerous amino-acid substitutions distributed throughout the proteins, variation in SmeRv was dominated by a limited number of highly divergent alleles containing large insertion/deletion events and extensive sequence differences. Despite this extensive sequence diversity, no individual amino-acid substitution or protein variant showed a consistent association with the co-trimoxazole non-wild type (NWT)/resistant phenotype across the entire dataset.

Phenicol resistance genes—*catB3* (*n* = 1), *cml* (*n* = 67), *cmx* (*n* = 3), *floR* (*n* = 2), and *floR2* (*n* = 1)—were identified in 71 of 323 (22.0%) isolates representing 10 species. Tetracycline efflux gene *tet(G)* was detected in a single isolate of *S. maltophilia*.

All isolates except those of Sgn4 harbored *qnr*-like genes encoding quinolone protection proteins: *qnrB* (*n* = 41), *qnrD* (*n* = 23), and *qnrE* (*n* = 257). Likewise, all isolates except two of *S. sepilia* carried *arr*-like genes encoding rifampin ADP-ribosylase.

### 3.6 Virulence genes

**Figure 5:**
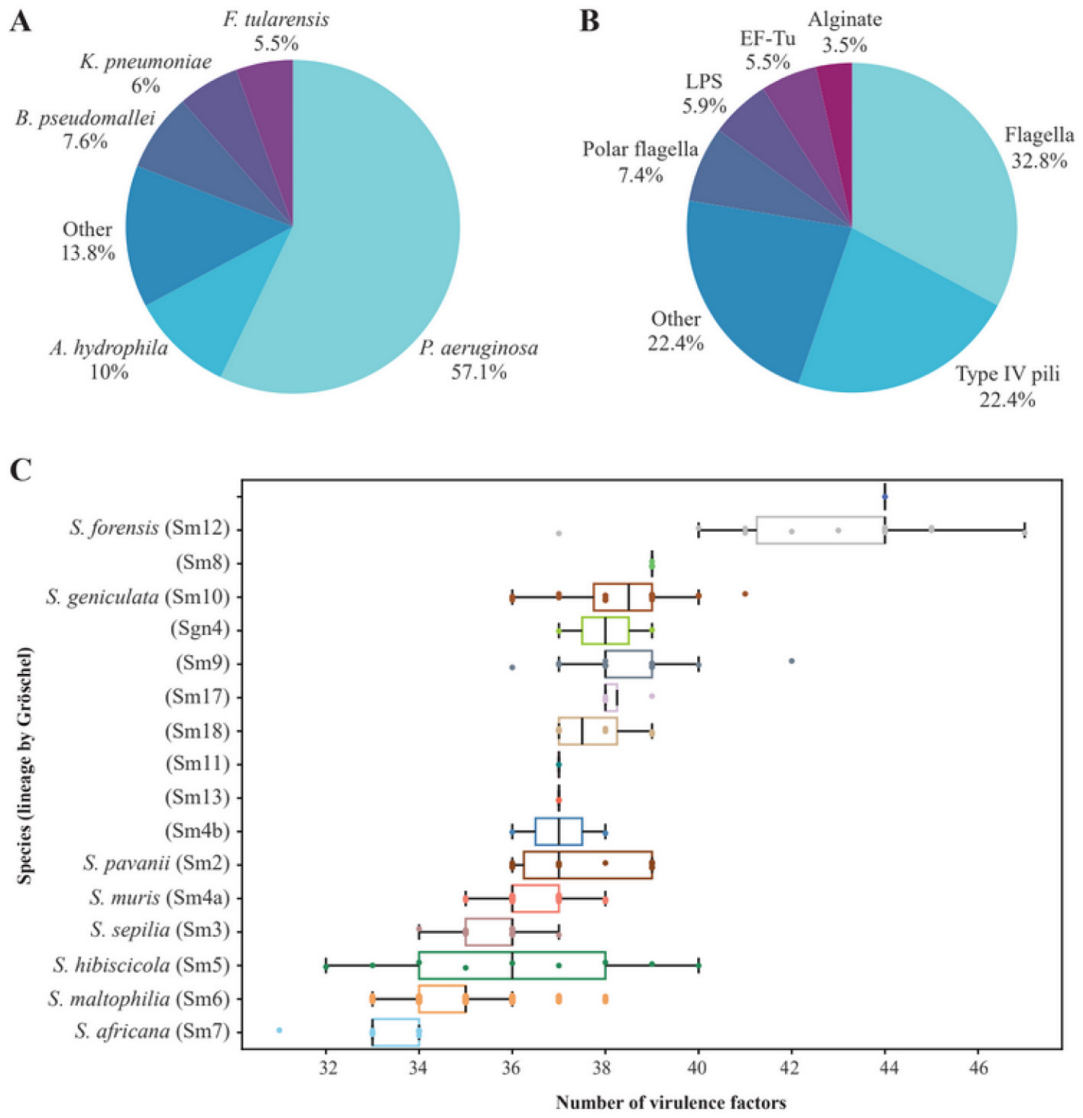
Virulence factors identified from VFDB across *Stenotrophomonas* spp. **(A)** Distribution of reference species with the highest identity to matched VFs. *P. aeruginosa, Pseudomonas aeruginosa*; *A. hydrophila, Aeromonas hydrophila*; *B. pseudomallei, Burkholderia pseudomallei*; *K. pneumoniae, Klebsiella pneumoniae*; *F. tularensis, Francisella tularensis*. **(B)** Functional classification of identified VFs. **(C)** Boxplot showing the number of VFs per species/lineage. Outliers and variation across lineages are indicated.

A total of 74 virulence factors (VFs) were identified using VFDB, including 24 genes present in nearly all analyzed genomes (Supplementary Table S7). However, only three genes—*pilG* and *pilT* (regulatory proteins of type IV pilus-mediated twitching motility), and *vipA* (type VI secretion system tubule-forming protein)—exhibited >80% identity to VFDB reference sequences. Notably, 57.12% of identified VFs, including these three, showed the highest similarity to genes from *Pseudomonas aeruginosa* PAO1, with the remainder matching various other gram-negative bacteria excluding *Stenotrophomonas*, reflecting the incomplete coverage of *Stenotrophomonas*-specific VFs in the VFDB database (Figure 5A).

The detected VFs spanned multiple functional classes, including flagella, type IV pili, elongation factor EF-Tu, capsule, lipopolysaccharide (LPS) O-antigen and alginate synthesis (Figure 5B). The median number of VFs per genome was 36 (range 31–47), with the highest counts in a single *S. riyadhensis* genome (*n* = 47) and in *S. forensis* genomes (range 37–47, median 44; Figure 5C).

**Figure 6:**
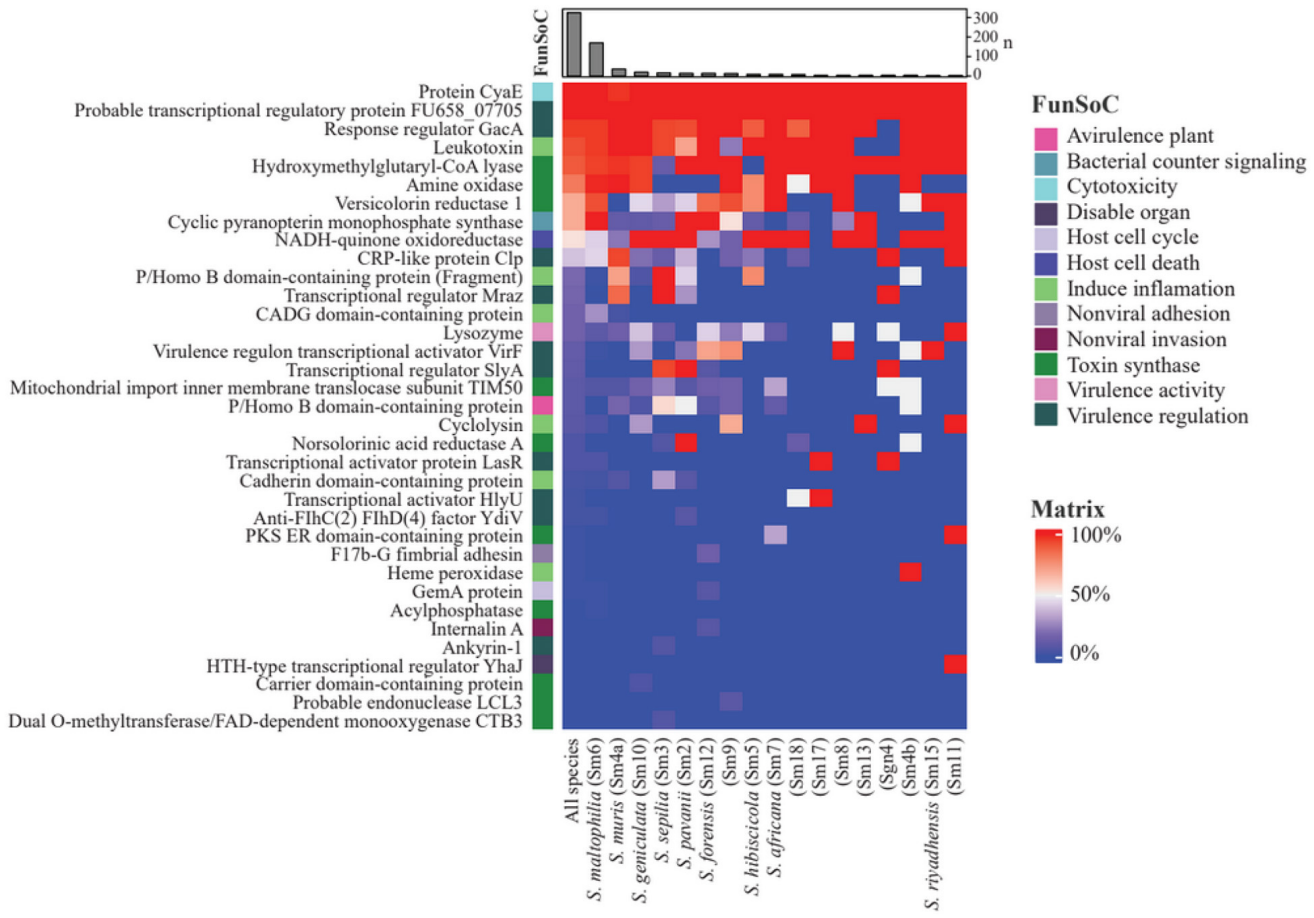
Heat map illustrating the prevalence of FunSoCs-associated genes across genomes of different species.

Additionally, the genomic pathogenicity potential was evaluated using SeqScreen, which employs a curated database mapping UniProt IDs to Functions of Sequences of Concern (FunSoCs) relevant to microbial pathogenesis. Twelve distinct FunSoCs, including virulence regulation, toxin synthesis, induction of inflammation, adhesion, invasion, and direct cytotoxicity, were associated with hits to 35 different genes (Figure 6). Among these, three genes—*cyaE* (protein export protein), FU658_07705 (probable transcriptional regulator), and *gacA* (response regulator of bacterial virulence gene expression), were identified in nearly all genomes analyzed. Most FunSoC-associated genes, however, exhibited variable presence across genomes from different species. Some, such as the Sm11 (isolate 107298) gene encoding a bacterial ankyrin-like protein implicated in host *ankyrin-1* disruption and organ dysfunction, were detected in only a single genome.

The median number of FunSoC-associated genes per genome was 9 (range: 6–13), with the highest counts (n = 15) in *S. pavanii* genomes and a single genome of Sm11 (isolate 107298).

Beyond the VFDB and SeqScreen/FunSoC analyses, which often identify putative virulence-associated homologs from other bacterial taxa rather than *Stenotrophomonas*-specific virulence genes, the genomes were also screened for previously experimentally validated virulence determinants native to *Stenotrophomonas*. These included the extracellular protease genes (*stmPr1, stmPr2*, and *stmPr3*), fimbrial attachment (*smf-1*), biofilm polysaccharide formation (*rmlA*), LPS biosynthesis (*spgM*), quorum sensing signaling (*rpfF*), and flagellar motility (*fliC*). Six of these genes (*stmPr1, stmPr2, stmPr3, smf-1, rmlA*, and *spgM*) were present in all genomes, whereas two displayed lineage-dependent distributions: *rpfF* was present in 232/323 (71.82%) genomes, including 95.24% *S. maltophilia* and all *S. africana, S. forensis, S. geniculata, S. hibiscicola*, Sm8, Sm9, Sm11, and Sgn4, but was absent in the remaining species; *fliC* was detected in 251/323 (77.71%) genomes and showed variable prevalence across lineages, with high presence (77.78%−100%) in most species, including *S. maltophilia* (83.33%), low presence in *S. geniculata* (5%), and absence in *S. riyadhensis*, Sm8, Sm9, and Sm11 (Supplementary Table S3).

### 3.7 Plasmids and prophages

Extrachromosomal circular contigs, presumed to represent plasmids (median geNomad plasmid score 0.9849; range 0.7535–0.9952), were identified in the genomes of 26 isolates (8.05%) spanning 10 species, with the highest prevalence in *S. maltophilia* (*n* = 16). The detected plasmids varied in length from 2,530 bp to 334,729 bp (median 75,531 bp; Supplementary Figures S7A, B). Replicon genes were detected in only four plasmids by comparison to known references: two identified as IncP replicon type, and one each as rep_cluster_156 and rep_cluster_995. MOB-Suite classified the majority of plasmids (23/26, 88.46%) as non-mobilizable, with two plasmids predicted as mobilizable and one as conjugative. Clustering analysis revealed several plasmid clusters with two clusters composed of more than two plasmids each. The largest cluster comprised eight non-mobilizable plasmids from five species: *S. maltophilia* (*n* = 4), Sm17 (*n* = 1), *S. muris* (*n* = 1), *S. hibiscicola* (*n* = 1), and Sm9 (*n* = 1; Supplementary Figure S7). To assess whether this pattern could be explained by sequencing batch effects, we examined sequencing metadata for all plasmid-positive isolates. The eight plasmids were detected in isolates from seven independent Oxford Nanopore sequencing experiments performed on seven different dates. Furthermore, the corresponding plasmid contigs showed robust sequencing depth support (long reads: 48.2 × -164.1 × , mean 90.3 × ; short reads: 134.4 × -167.1 × , mean 150.7 × ), with individual values listed in Supplementary Table S1.

Antibiotic resistance genes (ARGs) were identified on four plasmids, including one predicted to be conjugative. These ARGs comprised a streptothricin N-acetyltransferase gene (*sat2*), AGME genes [*aph(3*′*)-VIa, aph(3*″*)-Ib, aph(6)-Id, ant(2*″*)-Ia*], a chloramphenicol exporter gene (*cmx*), a sulfonamide-resistant dihydropteroate synthase gene (*sul1*), and a penicillinase gene (*bla*_OXA − 2_; Supplementary Table S8).

In total, 1,259 prophage regions were predicted across 323 analyzed genomes, with a median virus score of 0.95 (range 0.71–0.98). Most prophages (*n* = 979, 77.76%) were classified only at the class level as Caudoviricetes, while 13.42% (*n* = 169) were assigned to the *Inoviridae* family, and 8.82% (*n* = 11) to the *Autographiviridae* family. The number of prophages per genome ranged from 1 to 11, with a median of 4 (Supplementary Figure S9A; Supplementary Table S9). Six genomes—four *S. maltophilia* and two *S. forensis*—harbored the highest prophage counts (8–11). Notably, the genome of *S. maltophilia* isolate 121040 contained 11 prophage regions, three of which were predicted to be intact (Supplementary Figure S9B).

## 4 Discussion

This study presents a comprehensive phenotypic and genomic characterization of 323 clinical isolates of the genus *Stenotrophomonas* collected over two decades across Russia as part of a sentinel surveillance program (Kuzmenkov et al., 2021). While the isolates were obtained from diverse infection sites, more than half were recovered from respiratory samples, including a subset from CF patients, consistent with the well-established role of *Stenotrophomonas* as primarily pulmonary pathogens (Brooke, 2012). Core- and pan-genome analyses revealed substantial species-level diversity within the studied *Stenotrophomonas* isolates, exposing limitations of routine clinical species identification. By integrating antimicrobial susceptibility profiling with comprehensive genomic annotation, we mapped the distribution of resistance and virulence determinants alongside the mobile genetic elements that facilitate horizontal gene transfer (HGT), providing critical insights into the pathogenic potential and evolutionary epidemiology of the *Stenotrophomonas* genus.

A primary finding of this study is the pervasive misidentification of *Stenotrophomonas* species within clinical diagnostic workflows. Although two commercial MALDI-TOF MS systems initially identified all isolates as *S. maltophilia*, Average Nucleotide Identity (ANI) analysis revealed that only 52% belonged to *S. maltophilia* sensu stricto. The remaining isolates represented 16 distinct genomic species: eight validly published under the ICNP (representing 36.8% of all isolates) and eight “novel” lineages (11.2%) previously described by Gröschel et al. (2020). While these novel lineages are consistently recovered in global studies (Ortiz Álvarez et al., 2025; Yu and Wang, 2024; Thant et al., 2026a; Singh et al., 2023), they remain without official taxonomic designations. It is important to note that our results do not indicate an intrinsic inability of MALDI-TOF MS to distinguish species closely related to *S. maltophilia*; rather, they highlight a limitation of current reference-spectra databases, which lack comprehensive entries for recently described *Stenotrophomonas* species. For instance, the current Bruker MBT IVD and MBT Compass libraries contain only a few entries labeled as “*Stenotrophomonas maltophilia*,” “*Stenotrophomonas* sp.,” or “*Stenotrophomonas* sp3,” plus five environmental species not typically associated with human infection (*S. acidaminiphila, S. koreensis, S. nitritireducens, S. pictorum*, and *S. rhizophila*). It is often unclear whether entries designated “*S. maltophilia*” correspond to *S. maltophilia* sensu stricto or to closely related species under current taxonomic frameworks. Such diagnostic ambiguities do not merely represent taxonomic nuance, they actively impede outbreak investigations, epidemiological surveillance, and tailored clinical management (Vasileuskaya-Schulz et al., 2011). Accordingly, expanded reference libraries and systematic evaluation of MALDI-TOF MS performance for resolving clinically relevant *Stenotrophomonas* species are warranted. Because many current clinical reviews, guidance documents, recommendations and guidelines still treat *S. maltophilia* as a monolithic proxy for this diverse group (Tamma et al., 2024; Turnidge et al., 2025; Monardo et al., 2025; Clinical and Laboratory Standards Institute, 2026; European Committee on Antimicrobial Susceptibility Testing, 2026b), our results emphasize the need for updated taxonomic frameworks that reflect the true genomic and phenotypic variability of these pathogens.

The phylogenomic and pangenome analyses presented here demonstrate high genomic plasticity in human-associated *Stenotrophomonas* spp., a trait likely contributing to their ecological versatility and clinical resilience. Core genes—defined by a presence in at least 99% of isolates—represented less than 10% of the total pangenome. In contrast, the ‘cloud' genome dominated the genetic repertoire, with genes present in fewer than 15% of isolates comprising 83% of the total gene pool. Robust species-level clustering across both core and accessory genomes underscores the stability of these evolutionary lineages. Furthermore, the fact that the accessory genome UMAP projection closely mirrored the core phylogeny suggests that HGT is largely lineage-specific, with greater restriction between species than within them.

A distinctive strength of our study was the comprehensive assessment of phenotypic antibiotic susceptibility in clinical *Stenotrophomonas* isolates using the reference broth microdilution method, which enabled in-depth comparison of MIC distributions and resistance profiles across different *Stenotrophomonas* species. Notably, we observed a strikingly high prevalence of trimethoprim-sulfamethoxazole (TMP-SMX) resistance (40.2%), a concerning finding given its historical role as a first-line agent and a cornerstone of combination therapy for *S. maltophilia* (Sarzynski et al., 2022). This resistance profile was not confined to *S. maltophilia* but was consistently found across other abundant species within our collection. The observed resistance rate substantially exceeds those reported by European and North American surveillance programs, which typically cite frequencies between 5% and 15% (Sader et al., 2025; Dadashi et al., 2023; Gajdács and Urbán, 2019; Banar et al., 2023). Conversely, our findings align more closely with reports from certain Asian regions, such as China and India, where resistance has surpassed 20% (Hu et al., 2018; Kaur et al., 2015). The elevated prevalence of TMP-SMX resistance likely reflects a confluence of local antibiotic stewardship practices and inherent sample collection biases. Specifically, our observed resistance rates may be skewed by the clinical profile of the isolates, the majority of which were recovered from cases of severe or hospital-acquired infections and instances of empirical therapy failure. Conversely, screening isolates and those with an uncertain etiological role were excluded from the surveillance program, potentially concentrating resistant phenotypes within our cohort. Interestingly, only 17 of the 130 TMP-SMX-resistant isolates in our study, ten of which were identified as *S. maltophilia*, harbored known sulfonamide resistance genes (*sul1* and *sul2*). The low prevalence of *sul* genes in our resistant cohort (13.1%) suggests the predominance of alternative mechanisms. Previous work using in vitro resistance-selection experiments and mutation analysis in isogenic backgrounds has identified genetic changes associated with TMP-SMX resistance. Three studies implicated mutations in SmeT and SmeRv that upregulate the SmeDEF and SmeVWX multidrug efflux pumps as key contributors to resistance in *S. maltophilia* (Sánchez and Martínez, 2018; Ochoa-Sánchez et al., 2024; Boonyong et al., 2025). Two additional studies reported point mutations in chromosomal target genes—*folP* and *folA*, encoding dihydropteroate synthase and dihydrofolate reductase, respectively—that may account for resistance in clinical *S. maltophilia* and environmental *S. acidaminiphila* strains (Ochoa-Sánchez et al., 2024; Huang et al., 2018; Sánchez-Osuna et al., 2020). To investigate the clinical relevance of the mutations identified in experimental studies, we screened all genomes for amino-acid substitutions in FolA, FolP, SmeT and SmeRv proteins. FolP showed the highest allelic diversity, followed by FolA, SmeT, and SmeRv (107, 86, 71 and 29 protein variants, respectively). Variation in FolA, FolP, and SmeT was largely due to widespread single amino-acid substitutions, whereas SmeRv diversity was driven by a few highly divergent alleles with large indels. Importantly, despite this pronounced sequence heterogeneity, no single amino-acid substitution or protein variant exhibited a consistent association with the TMP-SMX resistance phenotype across the full dataset. This likely reflects methodological challenges in distinguishing resistance-causing changes from background variation in a phylogenetically diverse collection, as well as possible epistatic interactions among loci and contributions from additional, as-yet unidentified genes or mutations. Furthermore, the prevalence of highly divergent SmeRv alleles characterized by large indels indicates that regulatory-region variation may alter efflux regulation in ways not captured by single-site association tests. Together, these observations imply that, unlike single-gene resistance mechanisms mediated by horizontally acquired *sul* or *dfr* genes in Enterobacterales, TMP-SMX resistance in *Stenotrophomonas* is likely driven by diverse chromosomal changes. Comprehensive genotype–phenotype correlation will therefore require larger, species-stratified cohorts, rigorous functional validation of candidate alleles (e.g., in isogenic backgrounds), and integration of transcriptional and structural data to resolve which variants exert reproducible effects on TMP-SMX susceptibility. Regardless of the underlying molecular mechanisms, the high prevalence of resistance observed in our study necessitates a critical reassessment of TMP-SMX as a primary treatment for *Stenotrophomonas*. These findings align with recent clinical guidelines and expert recommendations, which have begun to pivot away from TMP-SMX in favor of more robust, evidence-based alternatives (Tamma et al., 2024; Turnidge et al., 2025). Among other agents, notable acquired resistance to levofloxacin was observed; 11 isolates (3.4%) exhibited MICs exceeding the ECOFF of 4 mg/L. Significantly, four of these isolates were recovered from patients with cystic fibrosis, likely reflecting the intense selective pressure resulting from the frequent use of fluoroquinolones in this specific population.

*S. maltophilia* is characterized by intrinsic resistance to nearly all β-lactam antibiotics, primarily mediated by the co-expression of two chromosomal β-lactamases: L1, a zinc-dependent molecular sub-class B3 metallo-β-lactamase (MBL) that hydrolyzes penicillins, cephalosporins, and carbapenems; and L2, a molecular class A serine β-lactamase that extends the resistance spectrum to include monobactams such as aztreonam (Brooke, 2012). The combination of aztreonam with avibactam—a diazabicyclooctane inhibitor—circumvents this resistance; avibactam effectively inhibits the L2 enzyme, while aztreonam remains intrinsically stable against the L1 MBL (Mojica et al., 2017). Consistent with this mechanism, we found that this combination inhibited all *Stenotrophomonas* isolates at aztreonam concentrations ≤ 8 mg/L (with avibactam fixed at 4 mg/L), yielding a mode MIC of 2/4 mg/L. Several studies have shown that *bla*_L1_ and *bla*_L2_ are almost universally present in *S. maltophilia* isolates, whereas their presence in related species is variable or even absent, yielding overall detection rates of 75%−88% for *bla*_L1_ and 22%−95% for *bla*_L2_, depending on the species cohort (Gröschel et al., 2020; Yamada et al., 2023; Yinsai et al., 2023; Pintor-Cora et al., 2025; Mojica et al., 2019). In our study, *bla*_L1_-like and *bla*_L2_-like genes were detected in nearly all isolates but exhibited substantially greater nucleotide sequence diversity than previously reported (Yamada et al., 2023; Mojica et al., 2019)—up to 20.39% for *bla*_L1_-like and 25.13% for *bla*_L2_-like genes—which may account for their frequent misidentification or failed detection in earlier studies. Phylogenetic analysis confirmed that these genes are orthologous across the *Stenotrophomonas* genus, as they were characterized by higher intra-species than inter-species conservation, with most species forming distinct monophyletic clades. On the other hand, several species yielded polyphyletic clades in the β-lactamase gene trees indicating discordance between gene and genome phylogenies. Although such topological incongruence alone does not constitute definitive proof of HGT or recombination, complementary analyses provided additional evidence for these processes: recombination detection (RDP) identified intragenic recombination signals in both *bla*_L1_- and *bla*_L2_-like sequences, implicating different *Stenotrophomonas* species as putative donors and resulting in chimeric β-lactamase alleles. Together, these observations suggest that HGT and recombination have contributed to the mosaic evolution of β-lactamase genes within the genus, potentially shaping their diversity and functional properties.

Additionally, we identified acquired β-lactamase genes—including class D penicillinases (*bla*_OXA_ variants) and class A ESBL (*bla*_GES − 7_)—across seven isolates from three species (*S. maltophilia, S. geniculata*, and *S. pavanii*). These were often associated with integrative conjugative or mobilizable elements (ICEs and IMEs) harboring aminoglycoside resistance genes and components of Type IV secretion systems, which underscores a significant potential for HGT and acquired resistance via mobile genetic elements within clinical *Stenotrophomonas* populations (Boncompagni et al., 2025).

Previous studies have reported the presence of phosphoethanolamine transferase (PET) genes homologous to mobile colistin resistance (*mcr*) genes in individual *S. maltophilia* isolates (Xie et al., 2021; Li et al., 2019). Here, we extended these observations by confirming that chromosomally encoded PETs structurally similar to the MCR*-5*-like and MCR*-8*-like lineages are broadly distributed across *S. maltophilia* and other *Stenotrophomonas* species, with the exception of several lineages in which *mcr-8*-like genes were not detected. This broader phylogenetic distribution suggests that chromosomal PET genes represent a conserved feature of the genus rather than a specific characteristic of individual isolates, further supported by their conserved chromosomal positioning across species. Despite the presence of PET homologs, their contribution to colistin resistance in *Stenotrophomonas* remains uncertain. Sequence similarity between chromosomal PET genes and mobile colistin resistance genes does not by itself demonstrate functional equivalence to clinically relevant *mcr* determinants. This is evidenced by our susceptibility testing data, showing broad colistin MIC distributions across all species (MIC_50_/MIC_90_ of 16/64 mg/L, with most isolates resistant), alongside prior work demonstrating highly heterogeneous susceptibility to colistin in *S. maltophilia* driven by complex, dynamic responses, including adaptive resistance and heteroresistance (Martínez-Servat et al., 2018). In this context, PETs are likely to represent only one component of a broader and still poorly understood network influencing polymyxin resistance in *Stenotrophomonas*, rather than acting as standalone resistance determinants.

The contribution of plasmids to HGT within the *Stenotrophomonas* genus remains poorly defined. Current evidence suggests that while *Stenotrophomonas* occasionally harbors small or, more rarely, larger plasmids, including Col-type and IncR replicons, it relies predominantly on chromosomal mechanisms for its key resistance traits. Furthermore, the genus-wide plasmidome, particularly within non-*maltophilia* lineages, is largely uncharacterized, representing a significant knowledge gap attributable to the historical scarcity of systematic, long-read sequencing studies (Brooke, 2021; Ogbolu et al., 2024). Utilizing long-read nanopore sequencing, we identified extrachromosomal circular contigs—ranging in size from 2.5 kb to over 334 kb—representing putative plasmids in the genomes of 26 isolates (8%). These plasmid-bearing isolates spanned 10 distinct species, highlighting the diverse distribution of extrachromosomal elements across the genus. Plasmid replicon genes were assigned to known incompatibility groups (IncP, rep cluster 156, and rep cluster 995) in only four instances. Resistance genes (ARGs) were localized to four plasmids, including one IncP replicon predicted to be conjugative. Notably, while most plasmid contigs were bioinformatically predicted to be non-mobilizable, we observed striking sequence identity among plasmids across divergent *Stenotrophomonas* lineages. For instance, the largest cluster consisted of eight nearly identical plasmids distributed across five distinct species, including *S. maltophilia*, Sm17, *S. muris, S. hibiscicola*, and Sm9. This widespread distribution suggests that these plasmids may traverse species boundaries via non-canonical pathways, such as mobilization *in trans* by co-resident conjugative elements, outer membrane vesicle-mediated transfer, or other cryptic mechanisms (Garcillán-Barcia et al., 2025). These findings imply that the horizontal mobility of the *Stenotrophomonas* plasmidome may be significantly underestimated by traditional sequence-based profiling, which relies exclusively on the presence of canonical mobilization machinery.

The high density of prophage regions identified in our collection, with a median of four per genome, and dominance of Caudoviricetes and Inoviridae in our dataset are consistent with previous work showing that temperate phages are common and phylogenetically diverse in *S. maltophilia* and related taxa (Thant et al., 2026a; Fang et al., 2023; Lee et al., 2014). Such abundant and taxonomically varied prophage cargo likely contributes to genome plasticity, HGT, and the acquisition of virulence- or fitness-associated traits in this opportunistic pathogen (Ortiz Álvarez et al., 2025; García et al., 2008).

Last but not least, the virulence gene analysis provided additional insights into the pathogenic potential of *Stenotrophomonas* species. Although our data confirms that the VFDB is not specific to *Stenotrophomonas*, the consistent detection of genes associated with type IV pili, flagella, lipopolysaccharide biosynthesis, and alginate production suggests the presence of conserved virulence-related traits across the genus. These functional categories correspond to biological systems previously implicated in adhesion, twitching motility, biofilm formation, host colonization, and environmental persistence in *Stenotrophomonas* spp. Notably, many predicted virulence determinants showed highest similarity to those described in *Pseudomonas aeruginosa*, which may reflect shared ecological niches or, potentially, historical HGT events (Bhaumik et al., 2024). Consequently, most VFDB matches identified in our study comprise cross-genus putative virulence-associated homologs rather than *Stenotrophomonas*-specific virulence factors. Importantly, however, our observation that *S. forensis* and *S. riyadhensis* genomes harbored comparatively high numbers of predicted virulence genes is consistent with emerging evidence that pathogenic potential in *Stenotrophomonas* is not restricted to *S. maltophilia* (Thant et al., 2026b). Consistent with this perspective, SeqScreen analysis identified multiple pathogenicity-associated functional signatures (FunSoCs), including categories related to virulence regulation, toxin-associated functions, and host inflammatory responses, further suggesting a diverse and potentially underrecognized pathogenic repertoire within the genus (Mikhailovich et al., 2024; Trifonova and Strateva, 2019). Furthermore, whereas the VFDB and SeqScreen/FunSoC analyses often identified cross-taxa virulence homologs, the targeted analysis of experimentally validated *Stenotrophomonas* virulence genes provides lineage-specific functional insight. Six genes—*stmPr1, stmPr2*, and *stmPr3* (extracellular proteases that degrade host matrix proteins and inactivate pulmonary antiproteases) (Windhorst et al., 2002), *smf-1* (fimbrial adhesin essential for initial attachment and biofilm formation) (de Oliveira-Garcia et al., 2003), *rmlA* (involved in exopolysaccharide/LPS intermediate synthesis, critical for biofilm integrity) (Zhuo et al., 2014), and *spgM* (LPS core biosynthesis; mutants are avirulent and serum-sensitive) (Zhuo et al., 2014; McKay et al., 2003)—were present in all 323 genomes. This universal conservation underscores their fundamental roles in tissue invasion, immune evasion, and biofilm establishment across *Stenotrophomonas* species. In contrast, *rpfF* (DSF quorum-sensing synthase) and *fliC* (flagellin) showed lineage-dependent distributions. *rpfF* was present in 71.8% genomes, including most *S. maltophilia* isolates and all representatives of *S. africana, S. forensis, S. geniculata, S. hibiscicola*, Sm8, Sm9, Sm11, and Sgn4, but was absent in the remaining species. *fliC* was detected in 77.7% genomes, with high prevalence in most species, including *S. maltophilia*, but low presence in *S. geniculata* and complete absence in *S. riyadhensis*, Sm8, Sm9, and Sm11. The variable presence of *rpfF* implies significant differences in cooperative cell-cell signaling and the potential for “social cheating” behavior (Huedo et al., 2018), whereas the absence of *fliC* suggests compromised swimming motility, adhesion, and biofilm formation in flagellin-deficient lineages (Wu et al., 2022). These genetic differences point to potentially divergent pathogenic strategies and environmental adaptations that warrant direct experimental confirmation in the relevant species. Interestingly, a recent study has demonstrated that a human bacteremic isolate of *S. muris* exhibited significantly higher virulence in a murine infection model than the *S. maltophilia* type strain, resulting in rapid mortality of infected mice (Liu et al., 2025). Together, these observations highlight the importance of integrating genomic predictions with experimental infection models to better delineate the pathogenic landscape of *Stenotrophomonas* spp.

Our study demonstrates that clinically relevant *Stenotrophomonas* species share resistance and virulence traits, yet they also exhibit extensive genomic and taxonomic heterogeneity, which has direct practical consequences beyond nomenclature. First, species level resolution matters for molecular diagnostics: it affects target selection, assay design, performance evaluation, and result interpretation. Reporting genetically diverse isolates under the single label “*S. maltophilia*” risks compromising diagnostic sensitivity and specificity, as well as downstream decision-making. Second, species differentiation is epidemiologically relevant. Because *Stenotrophomonas* are predominantly nosocomial, resolving species can improve outbreak detection, source attribution, and transmission inference, even when clinical phenotypes overlap. Third, we acknowledge that a single study, however large, may not reveal species-specific differences for every clinical trait. Nonetheless, adopting a genome-based taxonomy is a prerequisite for detecting such differences, since lumping data under a broad species label impedes retrospective analyses and future discovery. Emerging reports already suggest species-linked variation in clinically relevant traits, including differential susceptibility to newer antibiotics such as cefiderocol (Ortiz Álvarez et al., 2025; Sakr et al., 2025), and divergent virulence profiles (Liu et al., 2025), which underscores that taxonomic refinement can have genuine clinical import. In sum, our findings both highlight the functional commonalities across *Stenotrophomonas* and establish a genomic framework that, when integrated into clinical and surveillance workflows, will improve diagnostic precision and antimicrobial resistance monitoring and enable targeted, species stratified studies to identify which taxonomic distinctions translate into meaningful clinical and epidemiological consequences.

## Data Availability

All genome sequences (reads and genome assemblies) obtained in this study were deposited in the NCBI database under BioProject accession number PRJNA1285103. The phylogenomic clustering data and isolates' metadata are available as an open-access project at Microreact (https://microreact.org/project/clinical-stenotrophomonas). The Nextflow pipeline used to automate taxonomic species assignment is available at GitHub (https://github.com/valery-shap/tax-pipeline/).
