# Supplementary material for: Genomic phylogeny, taxonomic classification, genetic landscape, and antibiotic resistance of clinical Stenotrophomonas isolates
Source: Front Microbiol. 2026 Jul 13;17:1854839. doi: 10.3389/fmicb.2026.1854839 (PMC13402532; doi:10.3389/fmicb.2026.1854839)
Supplement: Supplementary file 1 [file Data_Sheet_1.pdf]

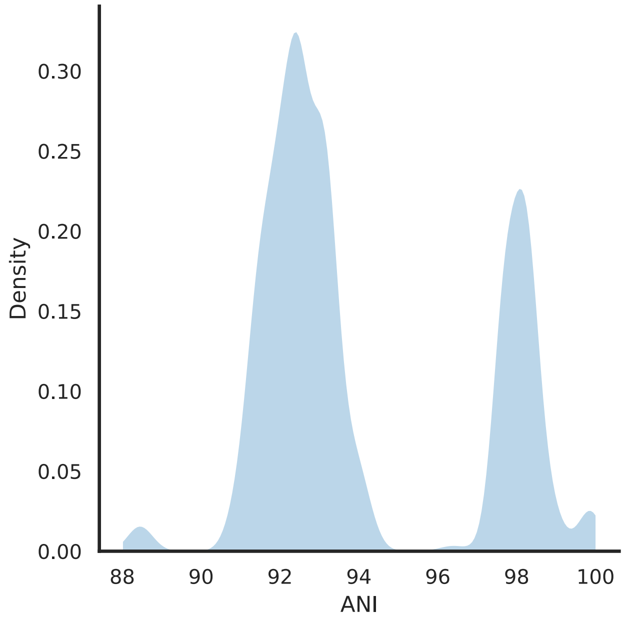

Supplementary Figure S1. Distribution of pairwise average nucleotide identity (ANI) values among analyzed genomes (n=323) of *Stenotrophomonas* species.

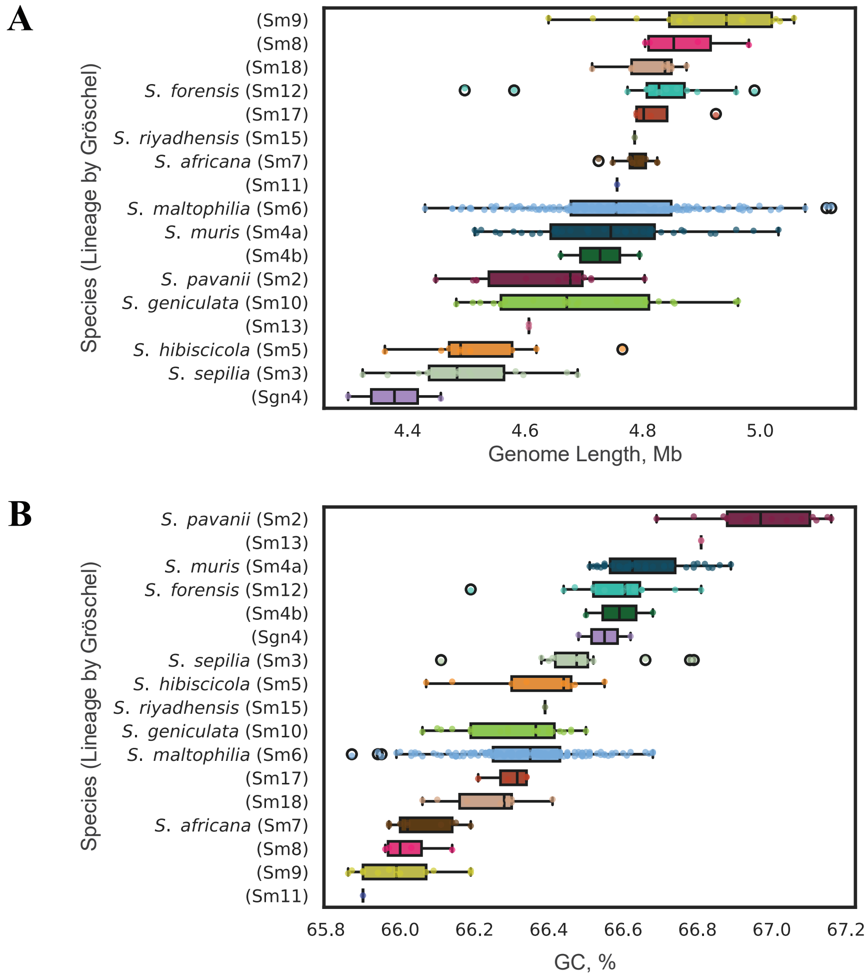

Supplementary Figure S2. Genome size (A) and GC content (B) distributions for each *Stenotrophomonas* species.

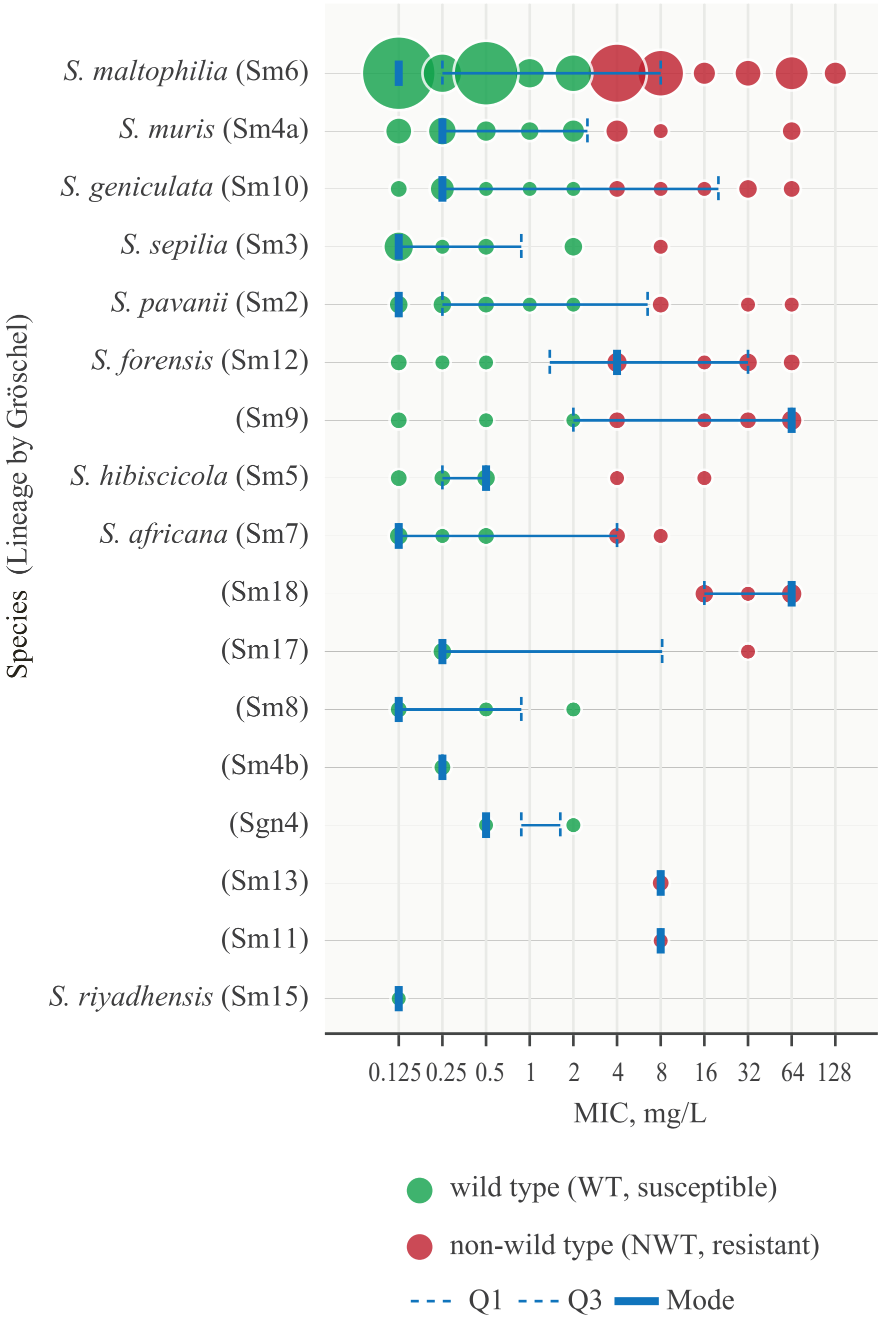

Supplementary Figure S3. Bubble plot of trimethoprim–sulfamethoxazole (TMP–SMX) MIC distributions across *Stenotrophomonas* species. Bubble size is proportional to the number of isolates.

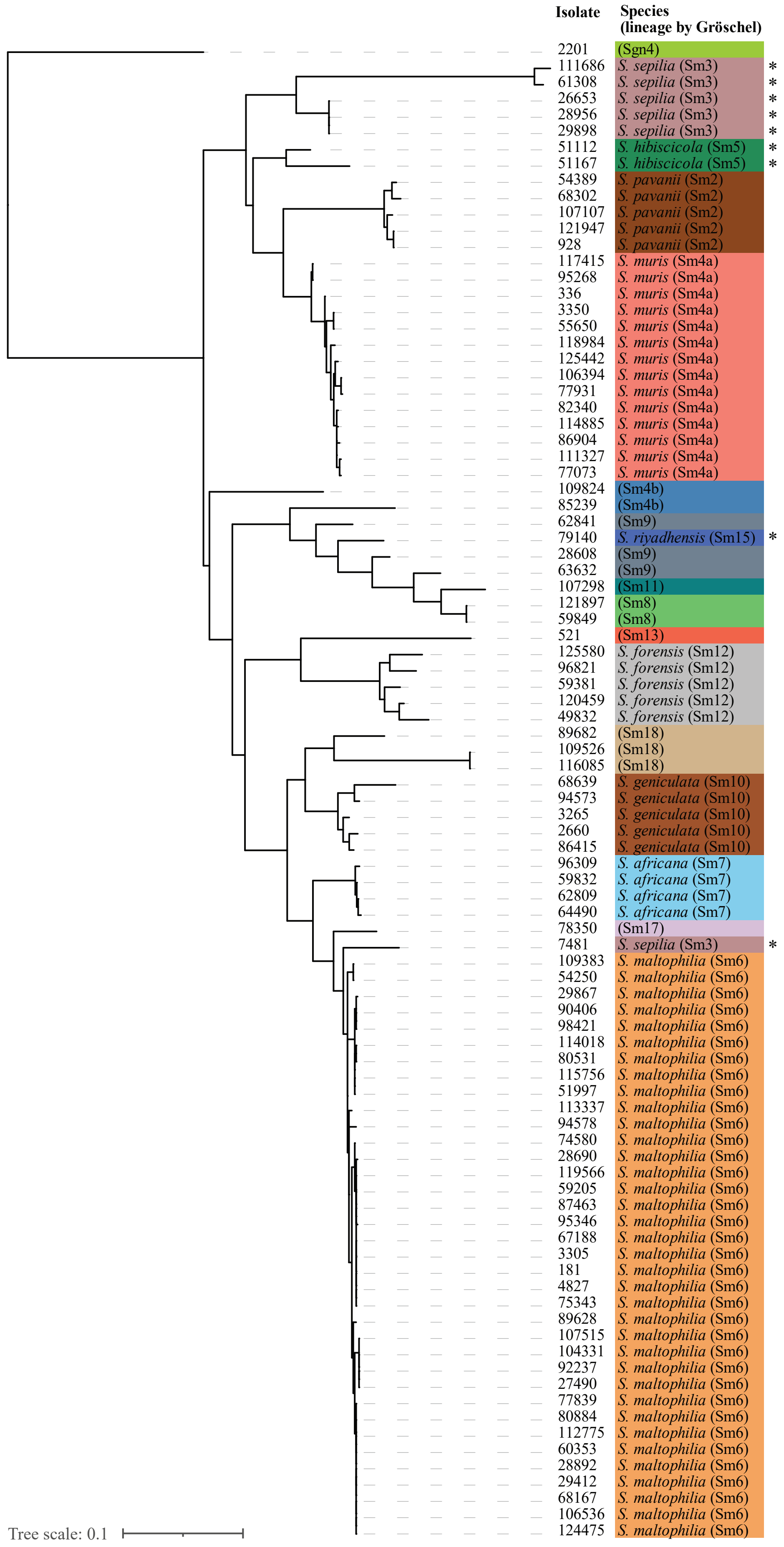

Supplementary Figure S4A. Maximum-likelihood phylogenetic tree of bla_{L1}-like genes from high-quality hybrid genome assemblies (n=92) visualized using iTOL.

Sequences showing evidence of potential interspecies recombination by Recombination Detection Program (RDP) analysis are marked with an asterisk (*).

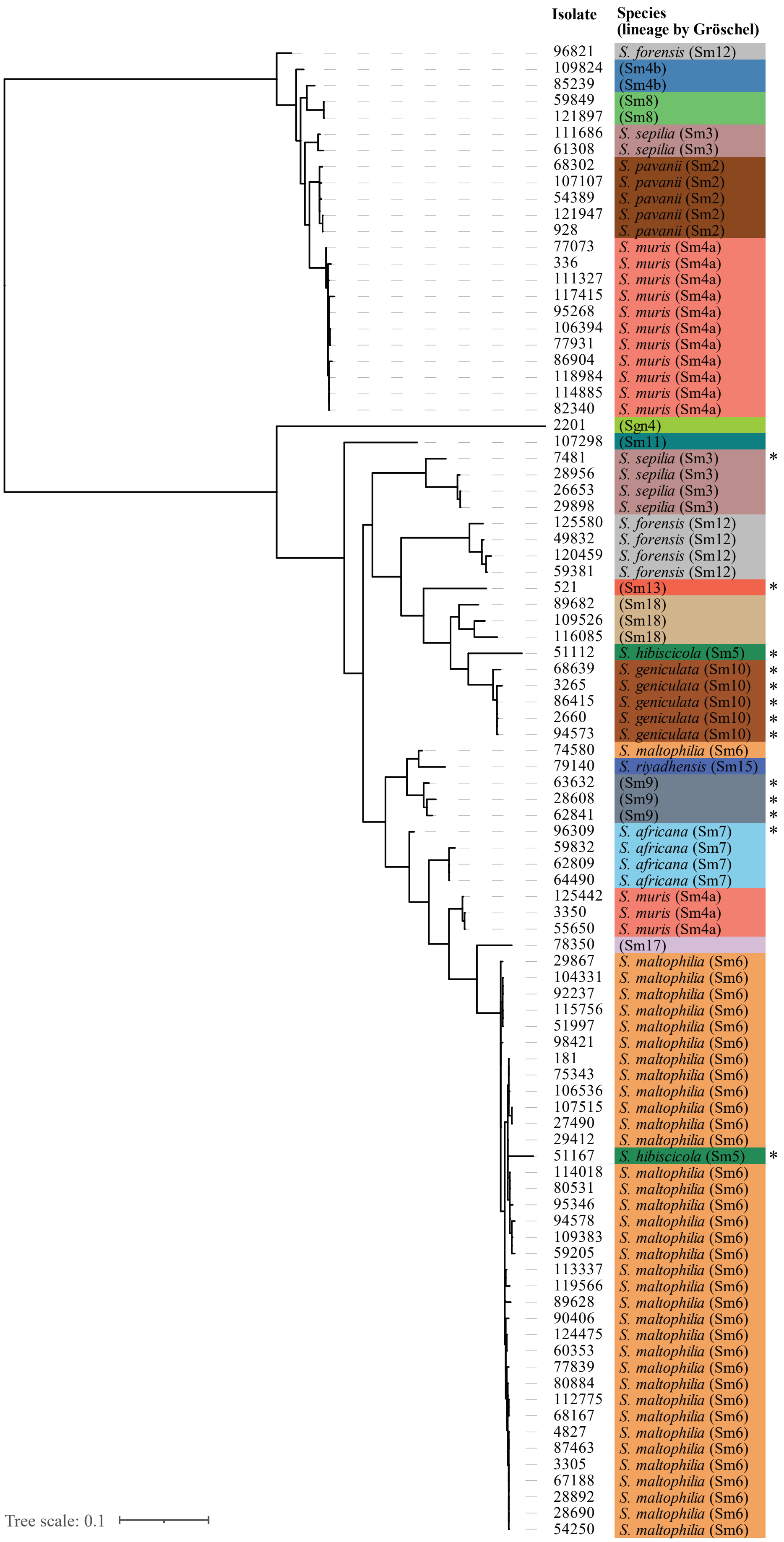

Supplementary Figure S4B. Maximum-likelihood phylogenetic tree of *bla*$_{L2}$-like genes from high-quality hybrid genome assemblies (n=92) visualized using iTOL.

Sequences showing evidence of potential interspecies recombination by Recombination Detection Program (RDP) analysis are marked with an asterisk (*).

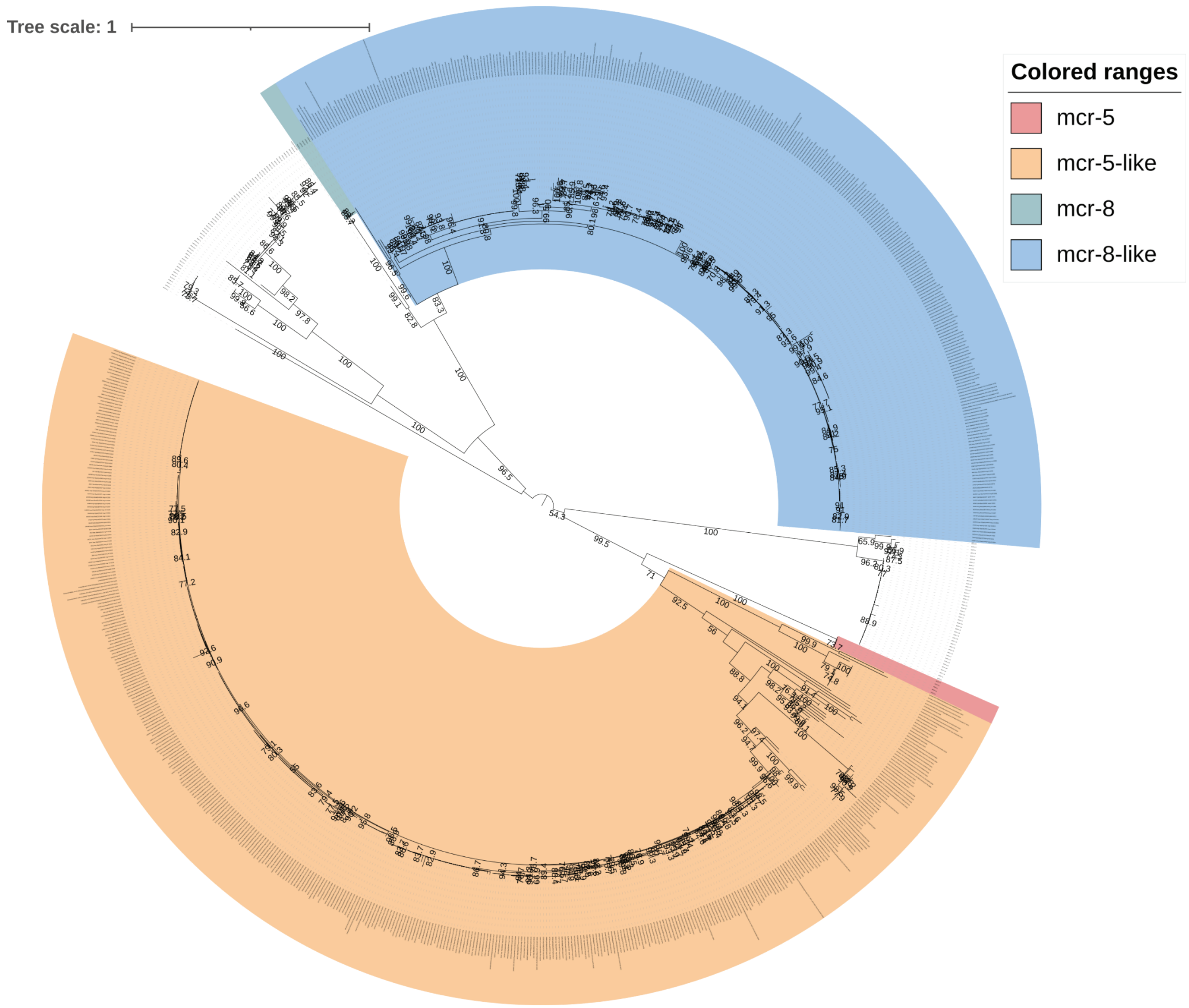

Supplementary Figure S5. Maximum-likelihood phylogeny of chromosomal phosphoethanolamine transferases (PETs) from *Stenotrophomonas* species and mobile colistin resistance (MCR) proteins.

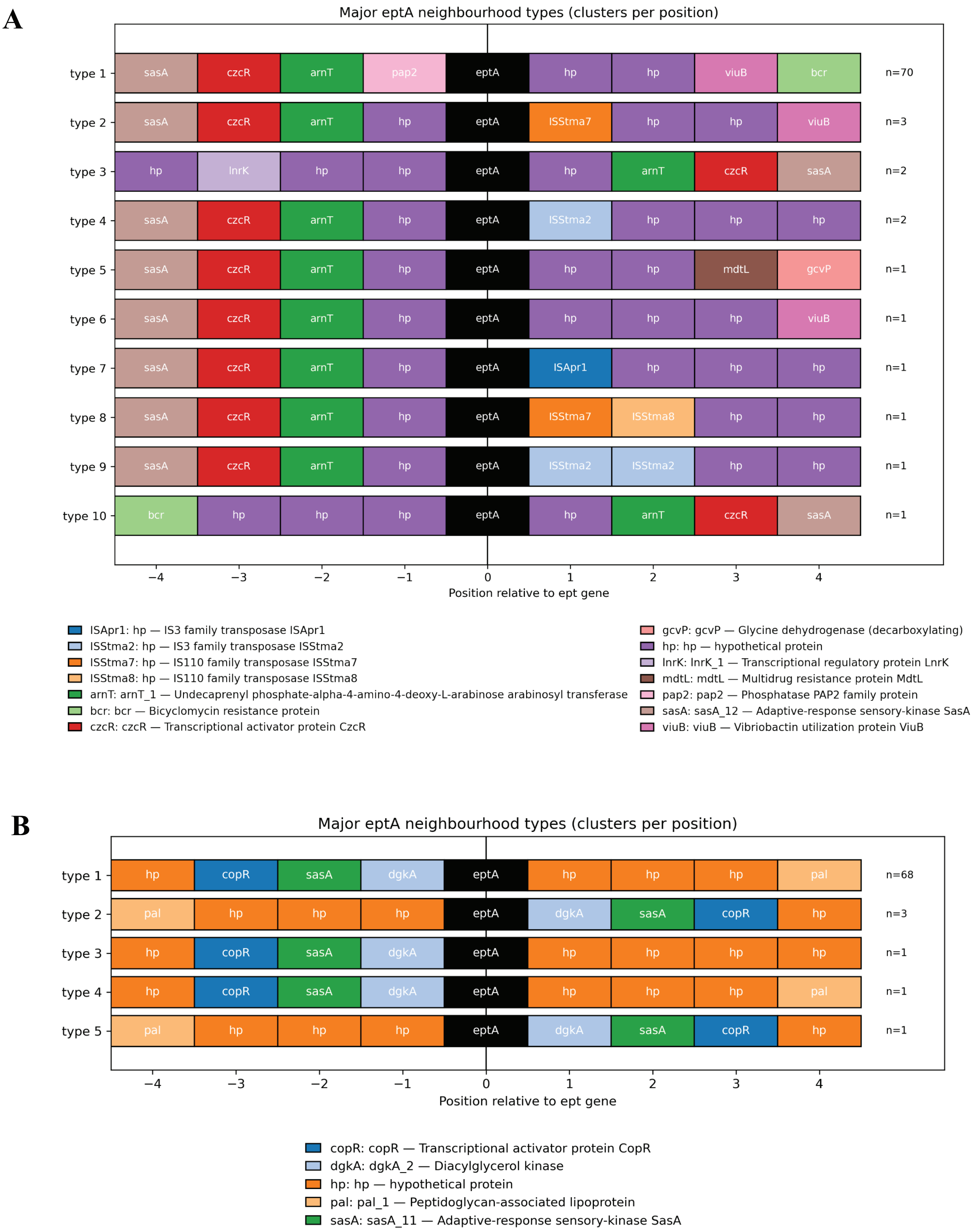

Supplementary Figure S6. Representative genomic neighborhoods of *eptA* genes encoding MCR-5-like (A) and MCR-8-like (B) PETs. Colored boxes denote genes within a ±4-gene window relative to *eptA* (black), with colors indicating functional annotation; “hp” denotes hypothetical proteins. Numbers on the right indicate the number of *eptA* loci associated with each neighborhood type.

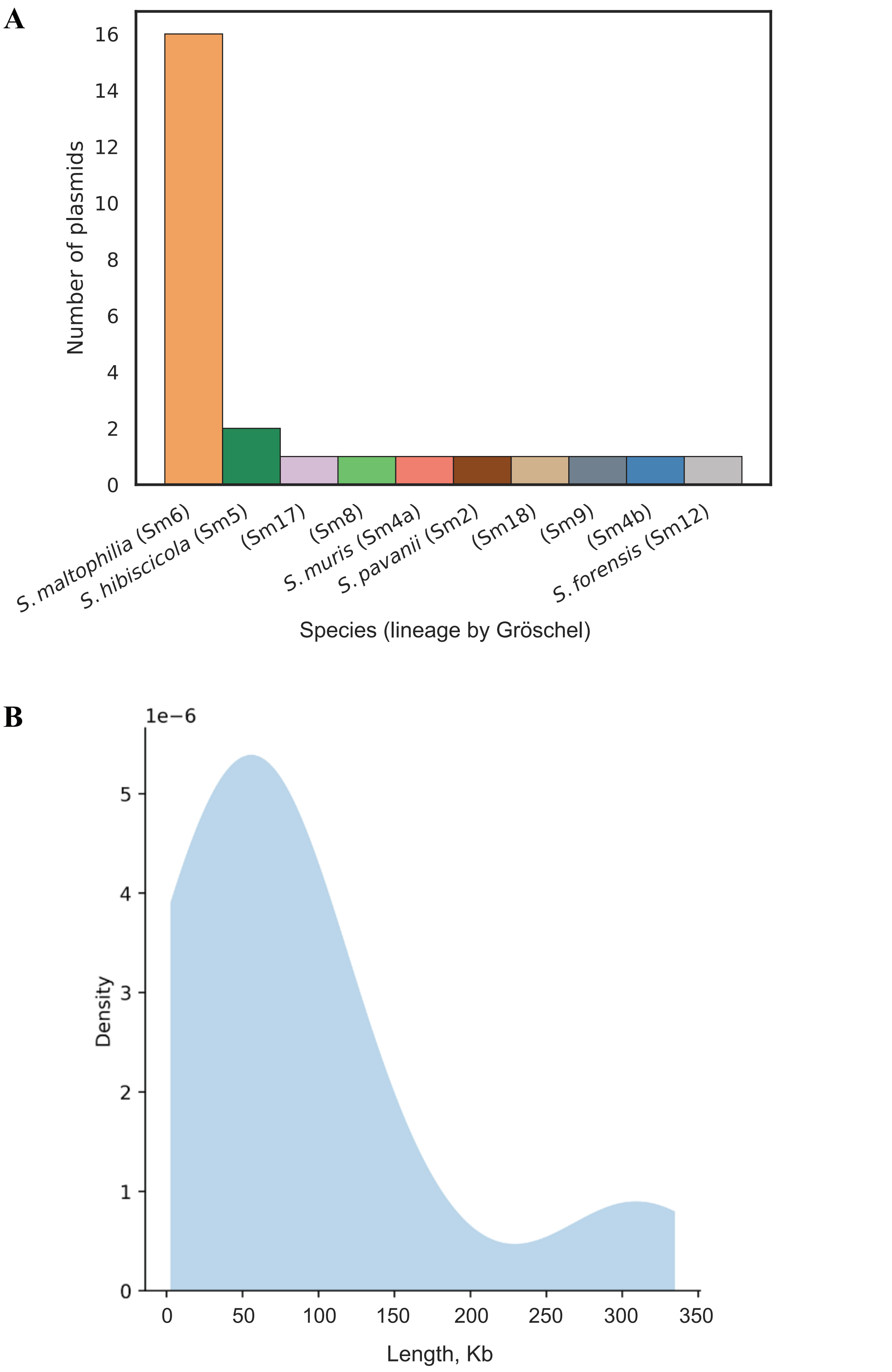

Supplementary Figure S7. Distribution of identified plasmid contigs by species (A) and size (B). The curve was drawn using a kernel density estimate.

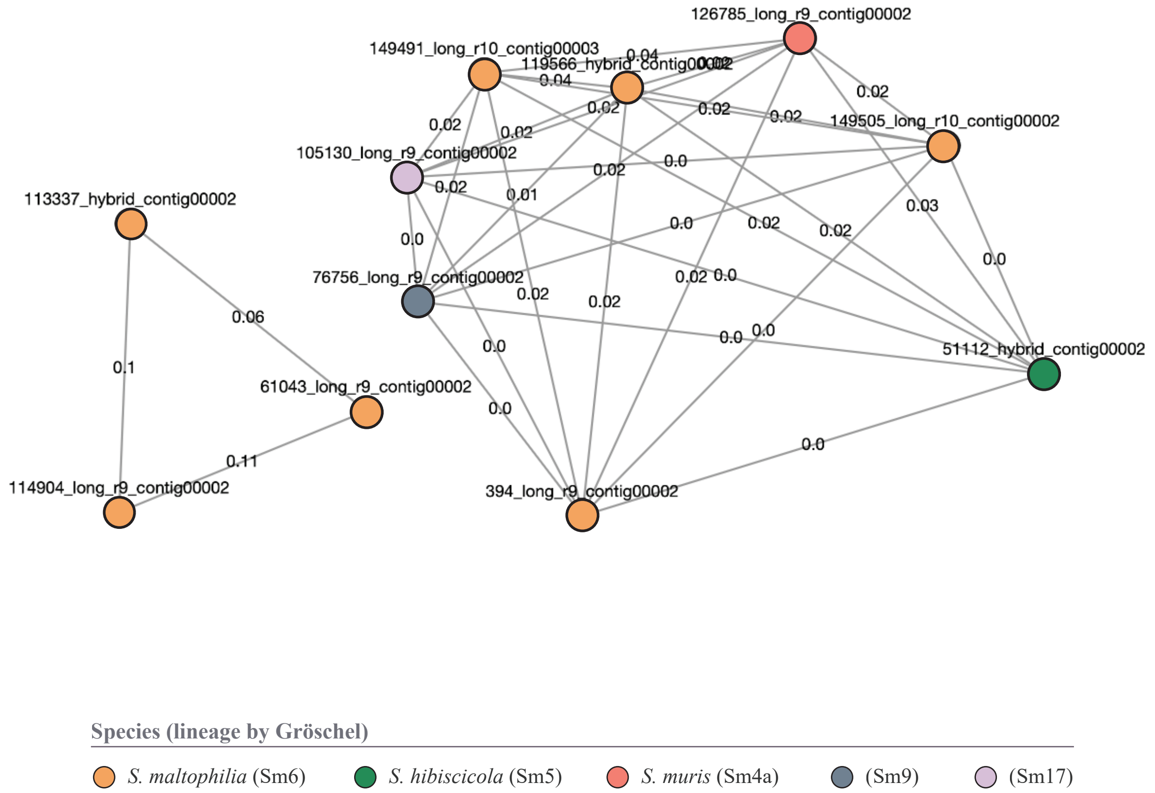

Supplementary Figure S8. Network clustering of plasmid contigs. Each node represents a plasmid sequence and is color-coded by species. Nodes connected by edges belong to the same plasmid cluster (community); numbers above edges indicate pairwise Double Cut and Join Indel (DCJ-Indel) distances.

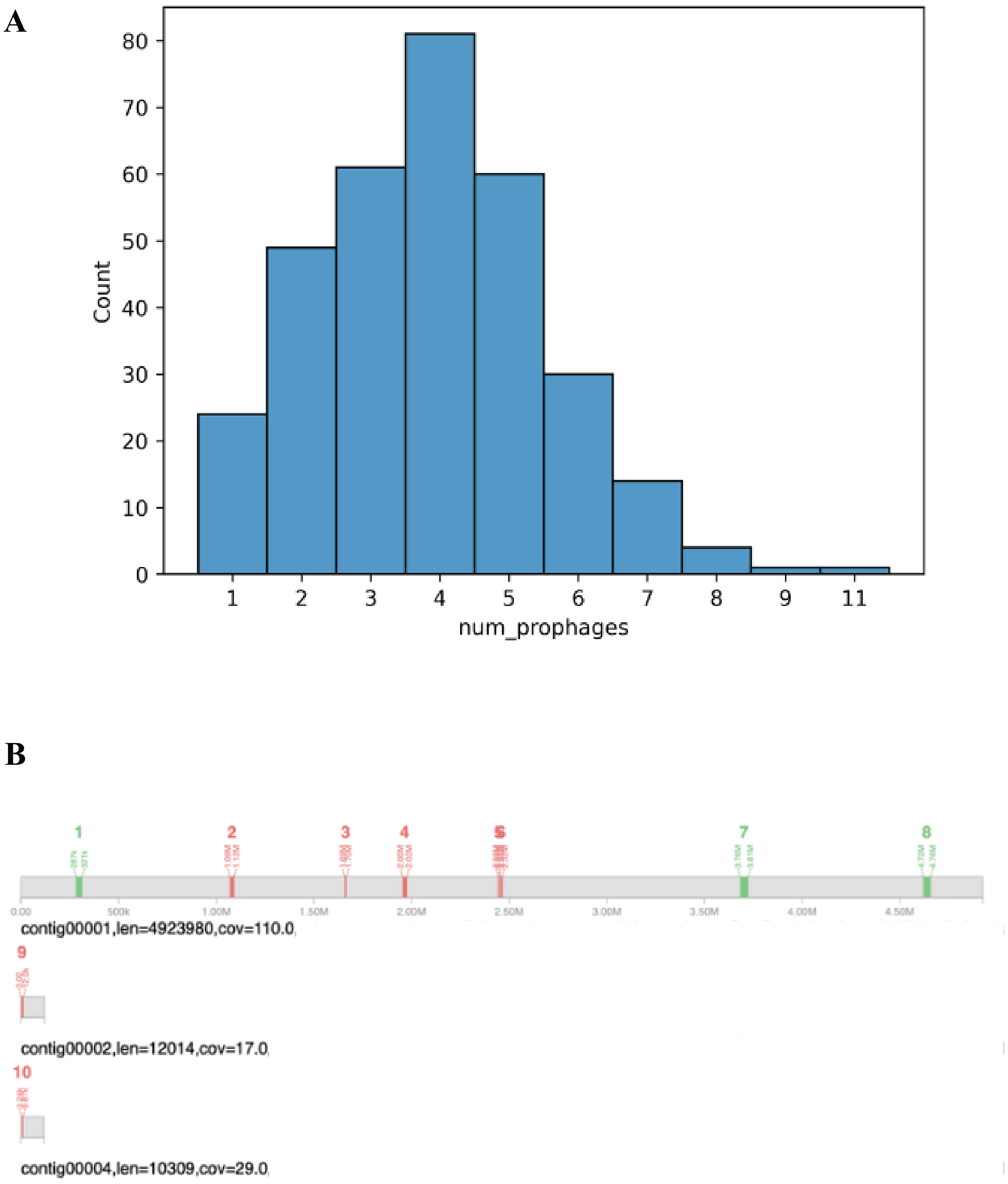

Supplementary Figure S9. Predicted prophage sequences in *Stenotrophomonas* genomes.
(A) Distribution of genomes by prophage count.
(B) Genomic localization of prophage regions in *S. maltophilia isolate* 121040, which harbors the highest number of prophages (n = 11). Only contigs >1,500 bp are shown. Intact regions (score >90) are marked in green, incomplete regions (score <70) in red.
